# ﻿Revisiting Bacan Island in the footsteps of Alfred Russel Wallace: unveiling the diversity of land snails (Mollusca, Gastropoda)

**DOI:** 10.3897/zookeys.1233.143563

**Published:** 2025-03-31

**Authors:** Ayu Savitri Nurinsiyah, Salma Syifa Azizah, Ahmad Ghifari Prasetia, Nova Mujiono, Ibnu Wahab Laitupa, Heryanto

**Affiliations:** 1 Research Centre for Biosystematics and Evolution, National Research and Innovation Agency, Jl. Raya Jakarta-Bogor Km 46, Cibinong, Indonesia Indonesian Malacological Society / Masyarakat Moluska Indonesia Bogor Indonesia; 2 Indonesian Malacological Society / Masyarakat Moluska Indonesia, Bogor, Indonesia Research Centre for Biosystematics and Evolution, National Research and Innovation Agency Cibinong Indonesia; 3 Department of Biology, Faculty of Mathematics and Natural Sciences, University of Jember, Jl. Kalimantan Tegalboto No.37, Jember, East Java, Indonesia University of Jember Jember Indonesia; 4 Department of Biology, Faculty of Mathematics and Natural Sciences, Universitas Padjadjaran, Jl. Ir. Soekarno Km 21, Hegarmanah, Sumedang, West Java, Indonesia Universitas Padjadjaran Sumedang Indonesia; 5 Universitas Muhammadiyah Maluku Utara, Jl. KHA. Dahlan, Kelurahan Sasa, Ternate, North Moluccas, Indonesia Universitas Muhammadiyah Maluku Utara Ternate Indonesia

**Keywords:** Biodiversity, Gastropoda, Indonesia, limestone, Maluku

## Abstract

There are a total of 47 species from ten families of Gastropoda recorded on Bacan Island from 1861 to 1963 by 15 studies. In 2022, the island was revisited to investigate its current land snail diversity. Our survey yielded 555 individuals, which were identified and classified into 27 species from eleven families. Among these, nine species were newly recorded on Bacan Island, bringing the total number of known land snail species to 56. These new records include a new species, *Dianctabatubacan* Nurinsiyah, Prasetia, Mujiono & Heryanto, **sp. nov.** The most abundant species collected was *Trochomorphaternatana* (family Trochomorphidae). Differences in sampling locations and the extent of forest habitats in the surveyed areas may account for the different number of recorded species from previous and recent studies. Comprehensive systematic and standardised surveys are crucial for ensuring sampling completeness to further assess species endemism and biogeographic patterns. Furthermore, cataloguing all known species and resolving land snail’s systematics with integrative approach are important to understand the true diversity of land snail in this region.

## ﻿Introduction

**Figure 1.**
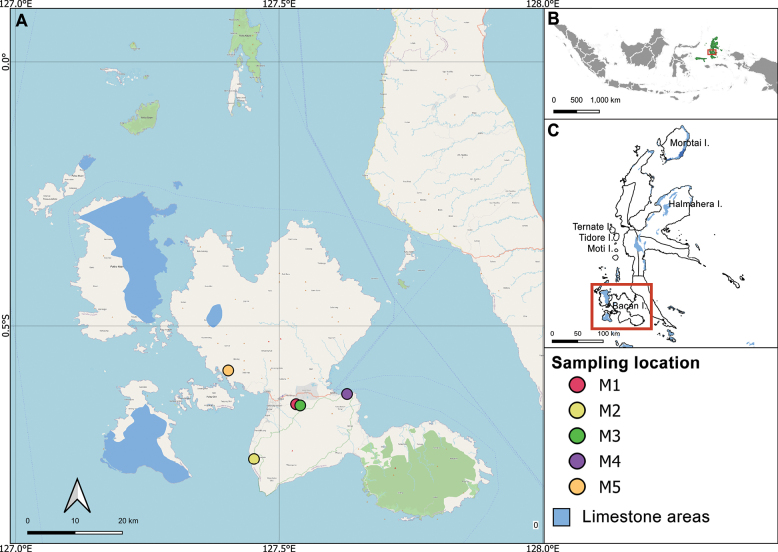
**A** map of Bacan Island and sampling locations of this study **B** map of Indonesia showing the location of Bacan Island (red square) **C** map of the North Moluccas Province showing the location of Bacan Island (red square).

Bacan Island is one of the major islands in the North Moluccas archipelago, Indonesia. It is part of the Wallacea region, which also includes the Moluccan islands, Nusa Tenggara, and Sulawesi. Administratively, Bacan Island belongs to the South Halmahera Regency in the Province of North Moluccas, with Labuha serving as its capital. The island spans an area of 2,792.85 km^2^ and has a population of 115,612 people (Badan Pusat Statistik Kabupaten Halmahera Selatan 2023). Geographically, Bacan is situated at the convergence of the Eurasian, Philippine Sea, and Australian tectonic plates, with Mount Batusibela (2,111 m a.s.l.) as its highest peak. The island’s oldest rocks, part of the Sibela Continental Suite, are believed to date back to the Precambrian era (Malaihollo and Hall 1996). The island can be divided into two primary ecological zones: (1) forested areas with emerging karst, and (2) non-karst areas such as forest fringes, the banana field, and the cocoa garden both with shrubs (Fig. 1).

Research on the biodiversity of Bacan Island has a long history, with one of the earliest studies conducted by Alfred Russel Wallace. From October 1858 to April 1859, Wallace stayed at Bacan Island, where he collected a large number of animal specimens (Wallace 1869). Several land snail specimens were also collected. Many of his collections were sent to collectors and museums in Europe. Hugh Cuming received the land snail specimens from Bacan Island collected by Wallace, which were later studied and published by Pfeiffer (1861). Pfeiffer described numerous species, including *Helixignescens* (= *Naninaignescens*), *Helixbatchianensis* (= *Trochomorphaternatana*), *Helixnodifera* (= *Papuinanodifera*), *Helixatrofusca* (= *Planispiraatrofusca*), amongst others. Wallace’s land snail collections were also preserved in the Melvill-Tomlin Collection. Notably, among these collections was the genus *Pyrochilus*, which is endemic to the islands of Gilolo, Ternate, and Bacan (Rowson and Wood 2015).

Edgar A. Smith (1896) published a list of land snails from the Moluccas, which included specimens from Bacan Island provided by Bruno Strubell. In total, Smith (1896) listed 43 species, among which 29 were recorded from Bacan Island. He described one new species from Bacan, Planispira (Cristigibba) lacteocincta. Later, Sykes (1904) described two additional new species from Bacan Island: *Obbasubgranulata* and *Papuinaecolorata*. The latter was synonymised with *Papuinavitrea* by van Benthem Jutting (1959). Sykes’ specimens were obtained from various sources, primarily through collections made by Fruhstorfer. Also in the same year, Gude (1904) provided a list of land snail species from Bacan Island which included 35 species. However, four species were later synonymised or excluded in the list of van Benthem Jutting (1959) for several reasons. The species *Albersiazonulata* Férussac 1821 and *Papuinagaberti*Lesson 1831 were previously recorded by Wallace (1865) as native to Papua, while their presence in the Moluccas remained uncertain (van Benthem Jutting 1959). The species Planispira (Cristigibba) anozona was synonymised with *Planispiraexpansa* (= *Cristigibbaexpansa* (Pfeiffer, 1861)) and *Planispirabuelowi*, Rolle 1903 was synonymised with *Planispiralacteocincta* Smith, 1896 by van Benthem Jutting (1959).

A comprehensive record of land snails from Bacan Island, especially from Labuha, Babang, and Wajaua, was compiled by van Benthem Jutting (1959) documenting 52 species including six variations i.e., Sulfurinaparvafa.electrina (Pfeiffer, 1859), Papuinapileolusvar.convexa (Férussac, 1821), Papuinapileolusvar.parabolica (Férussac, 1821), Papuinapileolusvar.furnita (Férussac, 1821), Planispira (Vulnus) endoptycha var. depressa Sykes, 1904, and Planispiraloxotropisvar.angusticlavia (Martens, 1867). Additionally, two species were synonymised (van Benthem Jutting 1959): *Cyclotusbernsteini* von Martens, 1863 with *Cyclotusbatchianensis* Pfeiffer, 1861 and *Cyclotuscodonostoma* Möllendorff, 1902 with *Cyclotusguttatus* (Pfeiffer, 1853). In 1963, Loosjes added *Euphaedusacumingianamoluccensis* in the species list from Bacan Island based on the collection of A.M.R. Wegner stored in the Zoologisches Museum of Amsterdam. In total, 47 land snail species are currently recognised from Bacan Island.

In 2022, we conducted a land snail expedition in Bacan Island. During this mission, we identified nine new records of land snails for the island. Among the new records we also discovered a new species from the island: *Dianctabatubacan* sp. nov. from the family Diplommatinidae. With the addition of the new records, including the new species, the total number of land snail species currently recorded on Bacan Island has increased to 56.

## ﻿Materials and methods

### ﻿Study sites

The study was conducted in five locations in Bacan Island (Fig. 1), divided into karst and non-karst areas. The non-karst areas included (M1) a banana plantation with shrubs, a flat environment in Marabose Village, 0°38.97'S, 127°31.91'E of 128 m a.s.l.; (M2) a cocoa garden with shrubs, another flat environment in Sawadai Village, 0°45.18'S, 127°27.12'E of 84 m a.s.l.; (M3) a forest edge not far from a small stream, slope in Babang Village, 0°39.09'S, 127°32.39'E of 45 m a.s.l. The karst areas included (M4) a forest on karst at the small hill of Patinti Strait, Babang Village, 0°37.80'S, 127°37.71'E of 46 m a.s.l.; and (M5) a forest on karst, slope in Sumae Village, 0°35.11'S, 127°24.19'E of 54 m a.s.l.

### ﻿Sample collection

Field work was conducted from May to June 2022. Both live snails and empty shells were carefully searched for by three persons for approximately five hours per sampling site, among leaf litter, rocks, wood debris, and plant stems. Live snails were preserved in 70% alcohol, while empty shells were stored in labelled plastic bags. All specimens are deposited in the Museum Zoologicum Bogoriense (**MZB**), National Research and Innovation Agency in Cibinong, West Java.

### ﻿Sample determination

Sample preparation was conducted at the Research Center for Biosystematics and Evolution, Soekarno Science and Technology Area. Species identification was conducted by referring to Martens (1867), Fulton (1899), Kobelt (1902), Neubert and Bouchet (2015), and Greķe (2017). Species systematics followed Molluscabase.org (2025). All collected species were photographed using a Nikon d3200 camera for macro snail (D > 5mm) and DMC5400 camera with L.A.S V4.13.0 software adapted to a Z6 APO (Leica Microsystems, Heerbrugg, Switzerland) for micro snail (D < 5mm). Shell characteristics for each species were described and measured with the measuring program in Leica M60 and a vernier caliper to the nearest 0.1 mm. The shell microsculpture of the new species was documented using a JEOL JSM-IT200 scanning electron microscope (SEM). The following abbreviations are used throughout the text:

**D** Shell diameter/width

**H** Shell height

**ha** height of aperture

**da** diameter/width of aperture

**MZB** Museum Zoologicum Bogoriense

**W** number of whorls

## ﻿Results and discussion

A total of 555 individuals were collected from Bacan Island, representing 27 species across 11 families. Of these, nine species were recorded for the first time on the island including one species, *Dianctabatubacan* sp. nov. which was identified as new to science. Combined with previous records from the literature, a total of 56 land snail species from 13 families are now known from Bacan Island (Table 1) with 13 species so far only recorded from the island.

**Table 1.** Comparative species list for Bacan Island, North Moluccas, Indonesia. Symbols – √: found, ×: not listed, *: so far only recorded in Bacan Island.

No.	Family	Species	Literature	This study	
				Number of individuals	Relative abundance (%)
1	Helicinidae	*Sulfurinaparva* (Sowerby II, 1842)	√^3,4,5,6,7,14^	22	4.0
2	Cyclophoridae	*Cyclotusbatchianensis* Pfeiffer, 1861	√^1,3,4,7,14^	11	2.0
3		*Cyclotusguttatus* (Pfeiffer, 1853)	√^1,3,4,5,6,7,8,14^	54	9.7
4		*Leptopomadecipiens* Pfeiffer, 1861	√^1,14^	×	×
5		*Leptopomaglobulosum* Pfeiffer, 1861*	√^1,7,14^	7	1.3
6		*Leptopomahalmahericum* Strubell, 1892	×	8	1.4
7		*Leptopomaleucorhaphe* von Martens, 1863	√^4,7,14^	12	2.2
8		*Leptopomamassena* (Lesson, 1831)	√^9,14^	×	×
9		*Leptopomapapuanum* Dohrn, 1862	√^3,5,14^	×	×
10		*Leptopomapulicarium* Pfeiffer, 1861*	√^1,14^	×	×
11		*Platyrapheplicosa* (von Martens, 1863)	√^4,7,14^	5	0.9
12	Diplommatinidae	*Dianctabatubacan* sp. nov.*	×	16	2.9
13		*Dianctatorta* Boettger, 1891*	√^6,7,14^	45	8.1
14		*Diplommatinaradiiformis* Preston, 1913	×	66	11.9
15	Pupinidae	*Moulinsiacylindrica* (Fulton, 1899)	√^14^	16	2.9
16		*Moulinsiasolitaria* von Martens, 1863	×	45	8.1
17		*Tylotoechuspfeifferianus* (Adams, 1869)*	√^3,7,14^	×	×
18	Veronicelloidae	*Laevicaulisalte* (Férussac, 1822)	√^13^	×	×
19	Charopidae	*Philalankakusana* (Aldrich, 1889)	×	9	1.9
20	Chronidae	*Kaliellascandens* (Cox, 1872)	×	1	0.2
21	Clausiliidae	*Phaedusacumingianamoluccensis* (von Martens, 1864)	√^15^	3	0.5
22	Trochomorphidae	*Trochomorphafroggatti* (Iredale, 1941)	√^3,4,5,7,11,14^	10	1.8
23		*Trochomorphaternatana* (Le Guillou, 1842)	√^1,3–9,11,14^	101	18.2
24	Mycrocystidae	*Lamprocystisambonica* Boettger, 1891	√^6,11^	8	1.4
25		*Lamprocystis* ‘Bacan 1’*	×	16	2.9
26	Ariophantidae	*Naninaignescens* (Pfeiffer, 1861)	√^1,3,4,7,11,14^	×	×
27		*Naninaluctuosa* Beck, 1837	√^3,5,11,14^	×	×
28		*Naninasulfurata* von Martens, 1864	√^2,4,7,11,14^	×	×
29	Helicidae	*Xestacitrina* (Linnaeus, 1758)	×	6	1.1
30	Camaenidae	*Cochlostylapubicepa* von Martens, 1864	√^2,4,7,8,11,14^	13	2.3
31		*Cristigibbacorniculum* (Hombron & Jacquinot, 1847)	√^3,5,7,11,14^	×	×
32		*Cristigibbaexpansa* (Pfeiffer, 1861)*	√^1–4,6–9,11,14^	29	5.2
33		*Landouriawinteriana* (Pfeiffer, 1842)	√^6,7,11,14^	×	×
34		*Obbasubgranulata* Sykes,1904*	√^10,14^	×	×
35		*Papuinalanceolata* (Pfeiffer, 1862)	√^14^	×	×
36		*Papuinanodifera* (Pfeiffer, 1861)*	√^3,7,11,14^	1	0.2
37		*Papuinaohlendorfii* Kobelt, 1897*	√^8,11,14^	×	×
38		*Papuinapileolus* (Férussac, 1821)	√^3,4,7,8,9,11,14^	7	1.3
39		*Papuinarhynchostoma* (Pfeiffer, 1861)*	√^1,3,4,7,11,14^	17	3.1
40		*Papuinavitrea* (Férussac, 1821)	√^5,10,14^	×	×
41		*Planispiraatrofusca* (Pfeiffer, 1861)	√^1,3,4,7,8,11,14^	×	×
42		*Planispirabiconvexa* (von Martens, 1864)	√^2,14^	×	×
43		*Planispiraexceptiuncula* (Férussac, 1823)	√^3,4,7,8,11,14^	×	×
44		*Planispirakurri* (Pfeiffer, 1848)	√^3,7,11,14^	×	×
45		*Planispiralacteocincta* Smith, 1896*	√^7,11,14^	×	×
46		*Planispiraloxotropis* (Pfeiffer, 1850)	√^4,7,11,14^	×	×
47		*Planispiraquadrifasciata* (Le Guillou, 1842)	×	2	0.4
48		*Planispirathetis* (Pfeiffer, 1851)	√^4,11,14^	×	×
49		*Planispirazonalis* (Férussac, 1821)	√^11,14^	×	×
50		*Planispirazonaria* (Linnaeus, 1767)	√^11,14^	×	×
51		*Pseudopapuinascheepmakeri* (Pfeiffer, 1850)	√^3,8,11,14^	×	×
52		*Pyrochiluspyrostoma* (Férussac, 1821)	√^7,11,14^	×	×
53		*Pyrochilussulcocinctus* (von Martens, 1865)	√^4,7,8,11,14^	×	×
54		*Pyrochilusxanthostoma* (von Martens, 1867)*	√^4,7,11,14^	×	×
55		*Sulcobasisconcisarubra* (Albers, 1857)	√^8,11,12,14^	×	×
56		*Vulnusendoptycha* (von Martens, 1864)	√^2,3,4,7,9,11,14^	25	4.5

^1^Pfeiffer (1861), ^2^von Martens (1864), ^3^Wallace and Adams (1865), ^4^von Martens (1867), ^5^Tapparone Canefri (1883), ^6^Boettger (1891), ^7^Smith (1896), ^8^Kobelt (1897), ^9^Fulton (1899), ^10^Sykes (1904), ^11^Gude (1904), ^12^Boettger (1914), ^13^Grimpe and Hoffmann (1925), ^14^van Benthem Jutting (1959), ^15^Loosjes (1963).

The family Cyclophoridae exhibited the greatest species richness, comprising six species. However, Diplommatinidae was the most abundant family, accounting for 127 individuals despite being represented by only three species, followed by Trochomorphidae accounting for 111 individuals from two species. Notably, *Trochomorphaternatana* (Trochomorphidae) was the most abundant species found in Bacan Island (18.2%). More than 50% of the collected species belong to the Caenogastropoda, a group of land snails often utilised as ecological indicators (Nurinsiyah et al. 2016).

A large difference in the number of specimens collected was observed across the five sampling locations and may be attributed to the land use (forested areas) in sites M3, M4, and M5 (Table 2). Specimens collected from M1 and M2 were fewer compared to those from M3, M4, and M5. The lowest abundance and species richness were observed at the cocoa garden sampling site (M2). However, the highest abundance was recorded in the forest non-karst area (M3) and the highest richness was recorded in the forest karst area (M5). Our findings revealed that forest habitats had more individuals and species (526 specimens and 27 species) than agricultural habitats (29 specimens and 5 species). The species composition (operculate and pulmonate species) was also higher in the forest area compared to agricultural areas both in richness and abundance. This suggests that forest ecosystems have higher population density and species richness than agricultural habitats. This result aligns with previous studies: Raheem et al. (2008) recorded 46 land snail species in forest area and 28 species in home gardens in Sri Lanka and indicated that habitat types have significant effects on land snail composition. Higher numbers of species richness in forest areas compare to agricultural areas were also demonstrated in studies in Java (Nurinsiyah et al. 2016; Nurhayati et al. 2021).

**Table 2.** Number of species abundance and richness in each sampling site.

	M1	M2	M3	M4	M5
	Non-karst area			Karst area	
	Agriculture/plantation		Forest		
**Number of species abundance**					
Abundance of operculate species	1	1	38	74	108
Abundance of pulmonate species	27	0	147	86	73
Total species abundance	28	1	185	160	181
**Number of species richness**					
Richness of operculate species	1	0	5	4	7
Richness of pulmonate species	3	1	4	6	10
Total species richness	4	1	9	10	17

Among the 27 species recorded on Bacan Island, the majority were distributed in humid areas containing karst formations. This pattern aligns with findings by Hausdorf (2019), who noted that land snails are more commonly found in volcanic soils and karst regions across various altitudes. This observation corresponds to the environmental characteristics of Bacan Island, which features extensive volcanic mountain ranges and karst forest areas. Our study reveals that the species abundance and species richness did not differ greatly between karst and non-karst areas. However, when considering habitat type, forest in karst area has the highest species richness. Previous studies indicated that the species richness and abundance in limestone areas were higher compared to non-limestone areas (Valdez et al. 2021; Boonmachai et al. 2024). These applied both for operculate and pulmonate species.

Differences in the richness of land snail species on Bacan Island can be attributed to several factors, with habitat alteration by humans being a critical driver. In particular, changes in forest cover on Bacan Island appears to have impact on land snail populations. However, even in the absence of direct human impact, the land snail biodiversity varies depending on various habitat characteristics, for instance soil moisture, soil pH, temperature, depth of leaf litter, canopy coverage, presence of deadwood, and the presence of herbaceous layers (Heryanto 2012; Douglas et al. 2013; Nurinsiyah et al. 2016; Rosales et al. 2020).

Land snails are known for their restricted geographic ranges and high sensitivity to habitat disturbances, including human activities, making them particularly vulnerable to local extinction (Nurinsiyah et al. 2016; Nurhayati et al. 2021; Boonmachai et al. 2024). The decline of land snail populations is often linked to their inability to adapt to extreme habitat changes. A notable indicator of habitat and community changes is the presence of micro-sized land snails. These species play a vital ecological role in the decomposition processes by facilitating fungal movement and contributing to nutrient cycling (Caldwell 1993; Pearce 2008). Micro-sized land snails are especially susceptible to environmental changes, particularly those caused by human disturbances (Douglas et al. 2013; Boonmachai et al. 2024).

The time elapsed since the earlier expeditions, such as those conducted in 1865, 1959, and 2017, highlights the need to compare the community structures of land snails in the recent and past forest conditions. The recorded diversity of land snails on Bacan Island likely represents only a small fraction of the biodiversity in Indonesia, particularly in the North Moluccas. Conducting more extensive research is essential to provide a more accurate estimation of species diversity and to identify the various environmental factors influencing biodiversity in the region. Furthermore, cataloguing all known species, resolving taxonomic ambiguities through integrative methods (e.g., molecular analysis, shell morphometrics, and anatomical studies), and stabilising species nomenclature will establish a robust foundation for understanding the true diversity of land snails on Bacan Island and the Moluccas Archipelago. Additionally, systematic and standardised surveys are essential to achieving sampling completeness. Such efforts are crucial for assessing species richness, endemism, and biogeographic patterns in the region.

### ﻿Systematics

#### ﻿Class Gastropoda Cuvier, 1795

**Subclass Caenogastropoda**

**Family Diplommatinidae L. Pfeiffer, 1857**

**Genus *Diancta* E. von Martens, 1864**

##### ﻿ Diancta batubacan

Taxon classificationAnimaliaArchitaenioglossaDiplommatinidae

Nurinsiyah, Prasetia, Mujiono & Heryanto sp. nov.

93C14AFA-9C62-5F7F-933B-65684F2F8BD5

https://zoobank.org/2AFDFC5E-4A8F-4BA7-B184-2C966DB3C03A

Fig. 2A–D

###### Type material.

***Holotype*.** Indonesia • Shell H = 5.2 mm; D = 2.8 mm; ha = 2.1 mm; da = 2.1 mm; W = 7.5; North Moluccas, Bacan Is., Sumae Village; 0°35.11'S, 127°24.19'E; alt. 54 m (M5); 1 June 2022; Heryanto, N. Mujiono, I.W. Laitupa leg.; MZB Gst. 23.855. ***Paratype*.** Indonesia • same locality as holotype; MZB Gst. 23.856/15. Both holotype and paratypes were deposited in the Museum Zoologicum Bogoriense (MZB).

###### Diagnosis.

Shell with bulbous penultimate whorl and a distinct constriction on the dorsal part of the penultimate whorl. Radial ribs are distinct before and after the constriction, but on the penultimate whorl the ribs are less distinct and almost smooth with no spiral striae. One outer parietalis, three parallel palatalis, and three perpendicular palatalis are present beside the constriction inside the penultimate whorl. Colour almost white.

**Figure 2.**
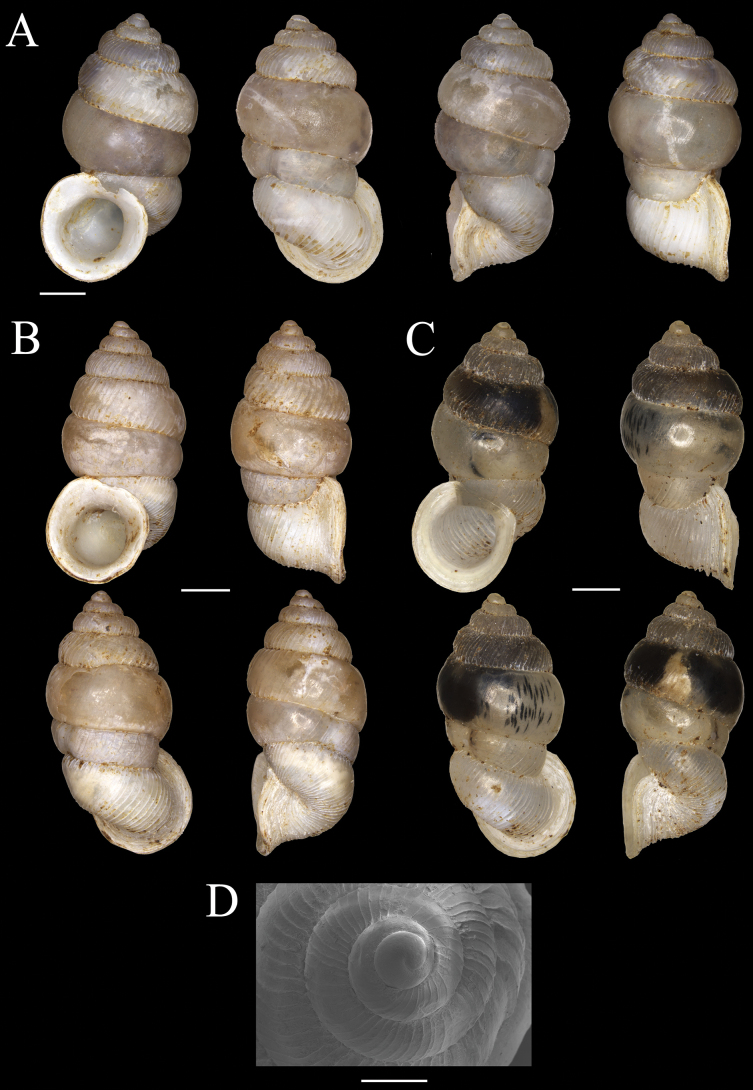
Shells of *Dianctabatubacan* sp. nov. **A** holotype (MZB Gst.23.855) **B, C** paratypes MZB Gst.23.856 **D** scanning Electron Microscope image apical view of paratype MZB Gst. 23.856. Scale bars: 1 mm (**A–C**); 0.4 mm (**D**).

###### Description.

The shell is small, elongated, and sinistral with a pointed apex, whitish cream in colour. The shell has 7–7.5 whorls that increase in size, with the body whorl being narrower than the penultimate whorl. There is a rapid increase in shell whorl size from the beginning of the teleoconch until the 5^th^ whorl, followed by a drastic constriction at the whorl 5.5–6, reducing the whorl size to that of the third whorl. The penultimate whorl (before constriction) is bulbous. The whorl size then increases again after the constriction for 1.5 whorls until the aperture. The umbilicus is closed. The protoconch is smooth, lacking ribs on the first whorl. Radial ribs are not sinuous but rather distinct, low, thin, and densely placed. The spacing between ribs increases from the beginning of the teleoconch until the 5^th^ or 6^th^ whorl, where the whorl size increases rapidly, causing the ribs to become less distinct and almost smooth. After the constriction, the ribs become more widely spaced before transitioning to a tighter spacing towards the aperture. Spiral striae are absent. A constriction is present on the penultimate whorl, with one outer parietalis, three parallel palatalis, and three perpendicular palatalis beside the constriction. The aperture is slightly oval and nearly rounded. Peristome simple because the outer and inner peristome fused. Operculum multispiral. Shell dimensions of the specimens (*n* = 16): H = 4.9–5.7 mm (mean 5.4 mm); D = 2.8–3.3 mm (mean 3.1 mm); ha = 2.0–2.4 mm (mean 2.2 mm); da = 1.8–2.3 mm (mean 2.1 mm).

###### Geographic distribution and habitat.

The species has so far been recorded on Bacan Island, making it possibly endemic to this region. Specimens were collected from the karst forest habitat.

###### Remarks.

Only one species of *Diancta* was previously recorded from Bacan Island, *Dianctatorta* Boettger, 1891 (Fig. 4D). The species differs from *D.batubacan* sp. nov. by having prominent and more radial ribs both in the penultimate and body whorl; the shell of *D.batubacan* sp. nov. has a smooth penultimate whorl (almost without ribs). The aperture of *D.torta* is tilted and oval, while the aperture in *D.batubacan* sp. nov. is more rounded. The shell of *D.batubacan* sp. nov. is similar to *D.halmaherica* Greķe, 2017 which is currently known only from Halmahera Island, and *D.constricta* which so far only found in Ternate and Tidore islands. Compared to *D.halmaherica*, *D.batubacan* sp. nov. has a more bulbous penultimate whorl, and the ribs on the penultimate whorl are less distinct. Additionally, the aperture of *D.batubacan* sp. nov. is not tilted. The constriction on the penultimate whorl of *D.halmaherica* is more pronounced. The shell of *D.batubacan* sp. nov. also differs with *D.constricta* in having less distinct ribs, no spiral striae, and has three parallel palatalis and three perpendicular palatalis inside the penultimate whorl.

###### Etymology.

The species name “batu bacan” refers to the famous Chrysocolla mineral/stone found on Bacan Island. Known for its striking beauty, the mineral shares a similar beauty to that of *Dianctabatubacan* sp. nov.

### ﻿Checklist

#### ﻿Class Gastropoda Cuvier, 1795

**Subclass Caenogastropoda Cox, 1960**

**Family Helicinidae Férussac, 1822**

**Genus *Sulfurina* Möllendorff, 1893**

##### *Sulfurinaparva* (Sowerby II, 1842)

Fig. 3A

**Type locality.** Philippine Islands.

**Material examined.** Indonesia • North Moluccas, Bacan Is., Babang Village; 0°37.80'S, 127°37.71'E; alt. 46 m (M4); 31 May 2022; Heryanto, N. Mujiono, I.W. Laitupa leg.; MZB Gst.22.926/22.

**Geographic distribution and habitat.***Sulfurinaparvaparva* (Sowerby II, 1842) and Sulfurinaparvaformaelectrina (Pfeiffer, 1859) were both recorded on Bacan and Halmahera islands. In addition, the former species was also recorded on Obi Island (van Benthem Jutting 1959). In this study, the species was found in karst forest.

**Figure 3.**
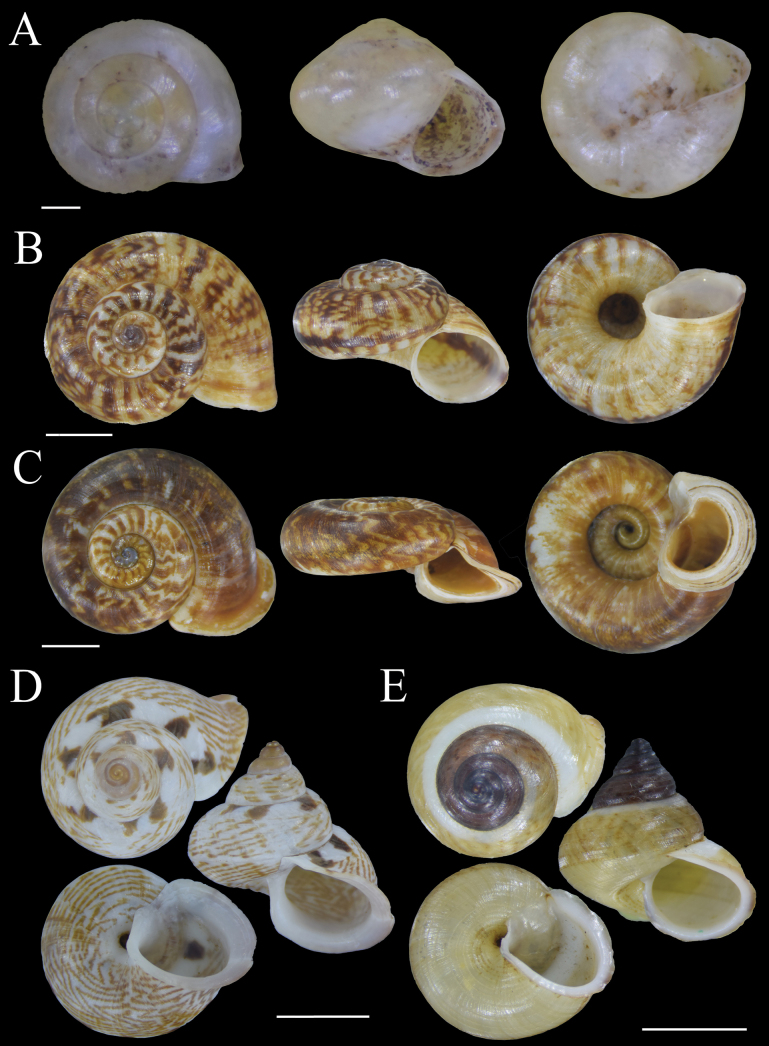
Shells of land snail species from Bacan Island **A***Sulfurinaparva* (Sowerby II, 1842) MZB Gst. 22.926 **B***Cyclotusguttatus* (Pfeiffer, 1853) MZB Gst. 22.914 **C***Cyclotusbatchianensis* Pfeiffer, 1861 MZB Gst. 22.915 **D***Leptopomahalmahericum* Strubell, 1892 MZB Gst. 23.941 **E***Leptopomaleucorhaphe* von Martens, 1863 MZB Gst. 22.927. Scale bars: 1 mm (**A**); 5 mm (**B–E**).

**Figure 4.**
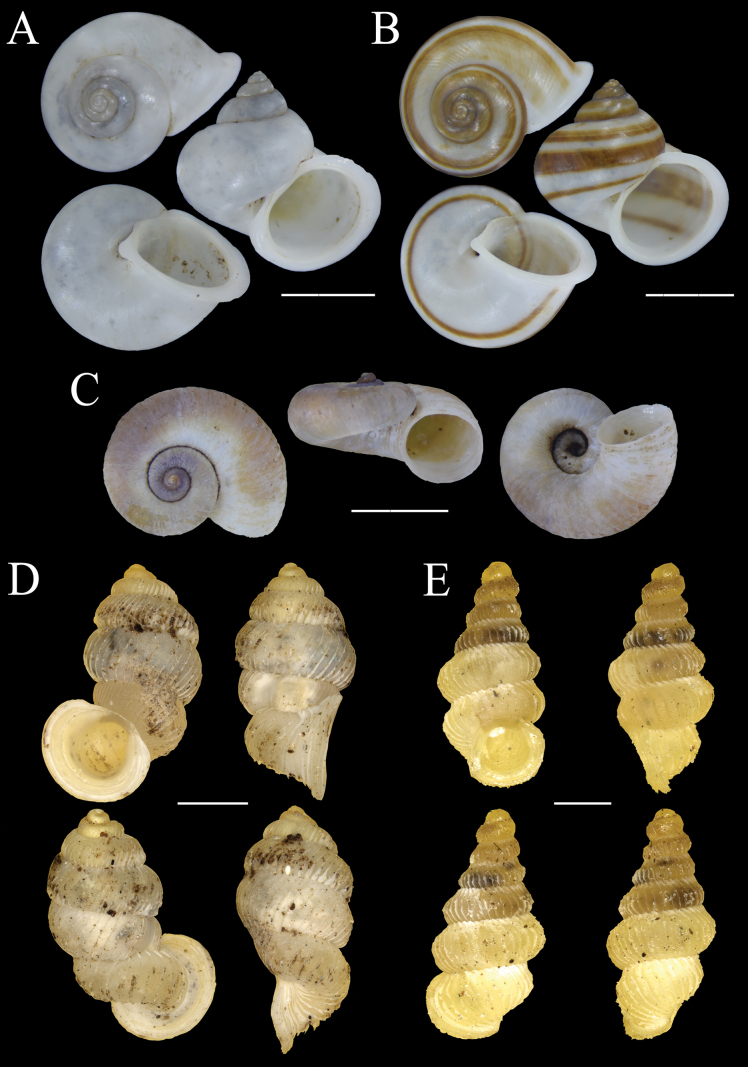
Shells of land snail species from Bacan Island **A, B***Leptopomaglobulosum* Pfeiffer, 1861 MZB Gst. 23.942 **C***Platyrapheplicosa* (von Martens, 1863) MZB Gst. 22.930 **D***Dianctatorta* Boettger, 1891 MZB Gst. 23.860 **E***Diplommatinaradiiformis* Preston, 1913 MZB Gst. 23.861. Scale bars: 5 mm (**A–C**); 1 mm (**D, E**).

**Description.** (*n* = 10) Shell with moderate size with H = 3.8–7.6 mm (mean 4.9 mm), D = 4.9–7.0 mm (mean 5.8 mm), ha = 2.1–3.0 mm (mean 2.3 mm), da = 1.3–2.5 mm (mean 1.9 mm), and whorl 4–5. Rounded - conical shell, yellowish white colour with a smooth and shiny surface. Umbilicus narrow, aperture quadrangular, peristome not continuous.

#### ﻿Family Cyclophoridae Gray, 1847

**Genus *Cyclotus* Swainson, 1840**

##### *Cyclotusbatchianensis* Pfeiffer, 1861

Fig. 3C

**Type locality.** Bacan Island.

**Material examined**. Indonesia • North Moluccas, Bacan Is., Babang Village; 0°39.09'S, 127°32.39'E; alt. 45 m (M3); 30 May 2022; Heryanto, N. Mujiono, I.W. Laitupa leg.; MZB Gst. 22.915/2• North Moluccas, Bacan Is., Sumae Village; 0°35.11'S, 127°24.19'E; alt. 54 m (M5); 1 June 2022; Heryanto, N. Mujiono, I.W. Laitupa leg.; MZB Gst. 22.929/9.

**Geographic distribution and habitat.** The species was so far only recorded on Bacan and Obi islands. In this study, the species was found in forest and karst area.

**Description.** (*n* = 10) Shell moderate size with H = 10.4–12.7 mm (mean 11.4 mm); D = 19.7–23.9 mm (mean 21.6 mm); ha = 5.9–7.8 mm (mean 6.5 mm); da = 5.3–6.9 mm (mean 6.2 mm); and whorl 4–4.5. Flat shell with bulging aperture, mottled brown colour, 4–4.5 whorls, last whorl large and rounded. Umbilicus open, aperture oblique, peristome continuous.

**Remarks.** The species was first described by Pfeiffer (1861: pl. III, fig. 1) as *Cyclotusbatchianensis*. *Pterocyclosbatchianensis* Pfeiffer, 1861 was later recorded on Bacan Island and placed in a different genus by Wallace (Wallace and Adams 1865), although no detailed description was provided. Möllendorff (1902) and van Benthem Jutting (1959) subsequently documented *Cyclotusbatchianensisbernsteini* von Martens, 1863 from Bacan and Obi islands. The shells of *C.batchianensis* and *C.pruinosus* von Martens, 1863 from Ternate, Tidore, Moti, and Halmahera islands are similar. However, resolving their systematics will require molecular phylogenetics and integrative taxonomy approach which is beyond the scope of this study.

##### *Cyclotusguttatus* (Pfeiffer, 1851)

Fig. 3B

**Type locality.** Unknown.

**Material examined**. Indonesia • North Moluccas, Bacan Is., Babang Village; 0°39.09'S, 127°32.39'E alt. 45 m (M3); 30 May 2022; Heryanto, N. Mujiono, I.W. Laitupa leg.; MZB Gst. 22.914/32• North Moluccas, Bacan Is., Sumae Village; 0°35.11'S, 127°24.19'E; alt. 54 m (M5); 1 June 2022; Heryanto, N. Mujiono, I.W. Laitupa leg.; MZB Gst. 22.930/22.

**Geographic distribution and habitat.** The species was recorded in Bacan Is., Ternate Is., and Halmahera Is. (van Benthem Jutting 1959). The species is distributed in the Wallacean region including North Moluccas (van Benthem Jutting 1959), Aru Island (Schepman 1919), and the northern part of Sulawesi (von Martens 1873). In this study, the species was found in the karst forest areas.

**Remarks.** Pfeiffer described the species in 1851 as *Cyclostomaguttatum* from the collection of Hugh Cuming. However, the type locality of this species was not mentioned. Later, Pfeiffer (1961) described *Cyclotussubflammulatus* from the collection of Alfred Russel Wallace on Bacan Island. Van Benthem Jutting (1959) synonymised the species to *Cyclotusguttatus*. Furthermore, Cyclotusguttatusformacodonostoma Möllendorff, 1902 was recorded in Bacan Island (van Benthem Jutting 1959). The subspecies has a more yellow-brown shell colour, the aperture double and slightly larger than *C.guttatus* with H = 14 mm and D = 22 mm (Möllendorff 1902).

**Description.** (*n* = 15) Shell large with H = 8.0–13.7 mm (mean 11.1 mm); D = 13.0–19.5 mm (mean 15.9 mm); ha = 5.0–7.7 mm (mean 6.5 mm); da = 5.0–7.8 mm (mean 6.3 mm); 4–4.5 whorls. The shell has a pyramidal shape, brown with yellowish white tones, last whorl large and rounded. Surface of shell textured. Umbilicus open, aperture oblique, peristome continuous.

#### ﻿Genus *Leptopoma* Pfeiffer, 1847

##### *Leptopomadecipiens* Pfeiffer, 1861

**Type locality**. Bacan Island.

**Remarks.** Not found in this study. This species was described from Bacan Island as *Leptopomadecipiens* and figured in Pfeiffer (1861: pl. III, fig. 10).

##### *Leptopomaglobulosum* Pfeiffer, 1861

Fig. 4A, B

**Type locality.** Bacan Island.

**Material examined**. Indonesia • North Moluccas, Bacan Is., Babang Village; 0°37.80'S, 127°37.71'E; alt. 46 m (M4); 31 May 2022; Heryanto, N. Mujiono, I.W. Laitupa leg.; MZB Gst. 22.925/4 • North Moluccas, Bacan Is., Sumae Village; 0°35.11'S, 127°24.19'E; alt. 54 m (M5); 1 June 2022; Heryanto, N. Mujiono, I.W. Laitupa leg.; MZB Gst. 23.942/8, 23.943/3.

**Geographic distribution and habitat.** So far, this species has only been found on Bacan Island. In this study, it was found at the forest edge in karst areas.

**Description.** (*n* = 11) Shell moderate size, H = 10.7–13.5 mm (mean 12.2 mm); D = 9.0–12.7 mm (mean 10.9 mm); ha = 5.3–7.7 mm (mean 6.3 mm); da = 5.0–6.7 mm (mean 5.9 mm); whorl 5–6.5. Conical shell with a pointed apex, transparent purplish-white in colour, sometimes with white base and brown stripes, last whorl large and convex. Umbilicus slightly open, aperture semicircle-oblique, peristome continuous. This species is said to be the same as *Leptopomavitreum*, but the difference between the two is the variation of shell colour and size (von Martens 1867).

**Remarks.***Leptopomavitreum*von Martens (1867) was described from the Moluccas region, in several forms, including those with a uniform colouration and those with brown spiral bands. In Bacan Island, most recorded specimens exhibited a uniformly white coloration, although individuals with brown spiral bands were also noted (von Martens 1867). However, a similar shell form from Bacan Island was previously described, *L.globulosum* Pfeiffer, 1861. The *Leptopoma* species show intra/interspecies variability and sexual dimorphism in shell shape and colour banding patterns (Phung et al. 2017, 2022). Geographical variation also influences shell characters in the *Leptopoma* (Phung et al. 2017). Based on this, we classify the *Leptopoma* species found in this study to *L.globulosum* and not *L.vitreum*. Phylogenetic analysis is required to confirm the taxonomic relationships of the two species and determine whether these forms represent intraspecific variation or distinct species.

##### *Leptopomahalmahericum* Strubell, 1892

Fig. 3D

**Type locality**. Halmahera Island.

**Material examined.** Indonesia • North Moluccas, Bacan Is., Babang Village; 0°39.09'S, 127°32.39'E alt. 45 m (M3); 30 May 2022; Heryanto, N. Mujiono, I.W. Laitupa leg.; MZB Gst. 22.913/2 • North Moluccas, Bacan Is., Sumae Village; 0°35.11'S, 127°24.19'E; alt. 54 m (M5); 1 June 2022; Heryanto, N. Mujiono, I.W. Laitupa leg.MZB Gst. 23.941/6.

**Geographic distribution and habitat.** New records for Bacan Island. The species was recorded on the Halmahera Is. (Greķe 2012). In this study, the species was found at the forest edge of the karst areas.

**Description.** (*n* = 8) Shell moderate size with H = 11.0–14.9 mm (mean 13.1 mm); D = 9.4–14.8 mm (mean 12.9 mm); ha = 5.2–7.8 mm (mean 6.5 mm); da = 4.3–7.8 mm (mean 6.3 mm); whorl 5–5.5. Conical shell with a pointed apex, white colour with brownish spotted pattern, and the last whorl large. Umbilicus closed, aperture oblique and looks like semicircle, peristome continuous.

##### *Leptopomaleucorhaphe* von Martens, 1863

Fig. 3E

**Type locality**. Halmahera Island.

**Material examined.** Indonesia • North Moluccas, Bacan Is., Sumae Village; 0°35.11'S, 127°24.19'E; alt. 54 m (M5); 1 June 2022; Heryanto, N. Mujiono, I.W. Laitupa leg.; MZB Gst. 22.927/12.

**Geographic distribution and habitat.** This species has been found in Halmahera and Kajoa islands (von Martens 1863) as well as South Halmahera and Bacan islands (van Benthem-Jutting 1959). In this study, the species was found in the karst forest areas.

**Description.** (*n* = 10) Shell large with H = 10.5–13.0 mm (mean 12.1 mm); D = 9.0–13.9 mm (mean 11.4 mm); ha = 4.0–7.7 mm (mean 5.4 mm); da = 4.1–6.6 mm (mean 5.1 mm); whorl 5–5.5. Conical shell with a pointed apex, brown with yellowish on the last whorl, 5–5.5 whorls, last whorl large and convex. Umbilicus slightly open, aperture oblique and semicircular, peristome continuous.

##### *Leptopomamassena* (Lesson, 1831)

**Type locality**. New Guinea.

**Remarks.** Not found in this study. This species was described as *Cyclostomamassena* from New Guinea. Subsequently van Benthem Jutting (1959) recorded the species from Bacan Island and placed it in the genus *Leptopoma*.

##### *Leptopomapapuanum* Dohrn, 1862

**Type locality**. New Guinea.

**Remarks.** Not found in this study. This species was described from New Guinea. Tapparone Canefri (1886) recorded the species from the Wallace collection on Bacan Island.

##### *Leptopomapulicarium* Pfeiffer, 1861

**Type locality.** Bacan Island.

**Remarks.** Not found in this study. This species was described from Bacan Island as *Leptopomapulicarium* and illustrated in Pfeiffer (1861: pl. 3, fig. 7).

#### ﻿Genus *Platyrhaphe* Möllendorff, 1890

##### *Platyrapheplicosa* (von Martens, 1863)

Fig. 4C

**Type locality**. Halmahera Island.

**Material examined**. Indonesia • North Moluccas, Bacan Is., Sumae Village; 0°35.11'S, 127°24.19'E; alt. 54 m (M5); 1 June 2022; Heryanto, N. Mujiono, I.W. Laitupa leg.; MZB Gst. 24.186/5.

**Description.** (*n* = 5) Shell small with H = 4.7–9.9 mm (mean 6.8 mm); D = 7.6–15.9 mm (mean 11.3 mm); ha = 3.6–6.0 mm (mean 4.8 mm); da = 3.1–5.7 mm (mean 4.5 mm); whorl 5–5.5. Flat shell with prominent apex, whitish colour, brown on apex, the shell has roughly textured lines. Umbilicus open, perpendicular aperture, peristome continuous.

**Remarks.** New record for Bacan Island. This species was described from Halmahera Island as *Cyclotusplicosus* and figured in von Martens (1867: pl. 2, figs 13, 14). In this study, it was collected in karst forest.

#### ﻿Family Diplommatinidae Pfeiffer, 1857

**Genus *Diancta* von Martens, 1864**

##### *Dianctatorta* Boettger, 1891

Fig. 4D

**Type locality**. Bacan Island.

**Material examined.** Indonesia • North Moluccas, Bacan Is., Babang Village; 0°37.80'S, 127°37.71'E; alt. 46 m (M4); 31 May 2022; Heryanto, N. Mujiono, I.W. Laitupa leg.; MZB Gst.23.858/13, MZB Gst.23.859/19, MZB Gst.23.860/13.

**Geographic distribution and habitat.** The species is so far only recorded on Bacan Island (van Benthem Jutting 1959; Greķe 2017). Thus, the species is possibly endemic to the island. In this study, the species was found in the karst forest.

**Description.** (*n* = 38) Shell small with H = 3.6–4.4 mm (mean 3.9 mm); D = 2.0–2.8 mm (mean 2.5 mm); ha = 1.1–1.8 mm (mean 1.6 mm); da = 1.1–1.8 mm (mean 1.6 mm); whorl = 6–7. Elongate and sinistral shell with pointed apex, cream whitish colour. Protoconch smooth without ribs for the 1.5 whorl. Teleoconch with dense distinct curved ribs. The ribs became distant on the body whorl towards the aperture. There is a constriction on the penultimate whorl. There are 6–6.5 whorls increasing in size, with body whorl narrower than penultimate whorl. Umbilicus closed, aperture oval to the left side, peristome expanded, thickened but not doubled.

#### ﻿Genus *Diplommatina* Benson, 1849

##### *Diplommatinaradiiformis* Preston, 1913

Fig. 4E

**Type locality**. Belang-belang Island.

**Material examined.** Indonesia • North Moluccas, Bacan Is., Babang Village; 0°39.09'S, 127°32.39'E alt. 45 m (M3); 30 May 2022; Heryanto, N. Mujiono, I.W. Laitupa leg.; MZB Gst. 23.861/66.

**Geographic distribution and habitat.** New record to Bacan Island. The species were recorded in the Moluccas archipelago i.e., Belang-belang Is. (Beilan-beilan Is.) to Obi Island (Preston 1913), Tidore Is., Halmahera Is. (Greķe 2017), and Moti Is. (Heryanto et al. 2023). In this study, the species was found at the edge of the forest and karst forest.

**Description.** (*n* = 10) Shell small size with H = 2.2–2.4 mm (mean 2.3 mm); D = 0.95–1.3 mm (mean 1.1 mm); ha = 0.6–0.7 mm (mean 0.7 mm); da = 0.7–0.9 mm (mean 0.8 mm); whorl = 7.5–8. Dextral shell and spindle shape with conical apex, whitish or corneous colour. Protoconch smooth, teleoconch with oblique ribs (penultimate and body whorl almost have same number of ribs) with spiral striae between ribs. The shell has 7.5–8 whorls increasing in size. Body whorl ventrally with constriction, on the inside with two spiral palatal folds close to suture. Umbilicus closed. Almost rounded aperture with distinct columellar lamella. Peristome expanded, thickened, and doubled.

#### ﻿Family Pupinidae Pfeiffer, 1853

**Genus *Moulinsia* Grateloup, 1840**

##### *Moulinsiacylindrica* (Fulton, 1899)

Fig. 5A

**Type locality.** Dodinga, Gilolo Island.

**Material examined.** Indonesia • North Moluccas, Bacan Is., Marabose Village; 0°38.97'S, 127°31.91'E; alt. 128 m (M1); 29 May 2022; Heryanto, N. Mujiono, I.W. Laitupa leg.; MZB Gst. 22.909/1• North Moluccas, Bacan Is., Babang Village; 0°37.80'S, 127°37.71'E E; alt. 46 m (M4); 31 May 2022; Heryanto, N. Mujiono, I.W. Laitupa leg.; MZB Gst. 24.600/15.

**Geographic distribution and habitat.** The species was recorded in Gilolo Island (now Halmahera Island) and Bacan Island (van Benthem Jutting 1959). In this study, the species was collected in banana field.

**Description.** (*n* = 5) The species was described by Fulton (1899) as Pupina (Moulinsia) cylindrica. Shell dextral, medium-sized for the genus with size H = 4.8–5.8 mm (mean 5.4 mm); D = 2.6–3.3 mm (mean 3.0 mm); ha = 1.9–2.1 mm (mean 2.0 mm); da = 1.7–2.2 mm (mean 1.9 mm) and whorl 5.5. The shell is brown and glossy, covers with minute nodules. The apex is obtuse rather than pointed. The last whorl constitutes ~ 3/5 of the total shell height. The umbilicus is closed, and the aperture is rounded-oblique. Aperture lip is thickened, and the peristome is not continuous. Parietal tooth and parietal callus are absent. A small and vivid perpendicular columellar tooth present, creating a little anterior canal. Posterior canal is absent.

**Figure 5.**
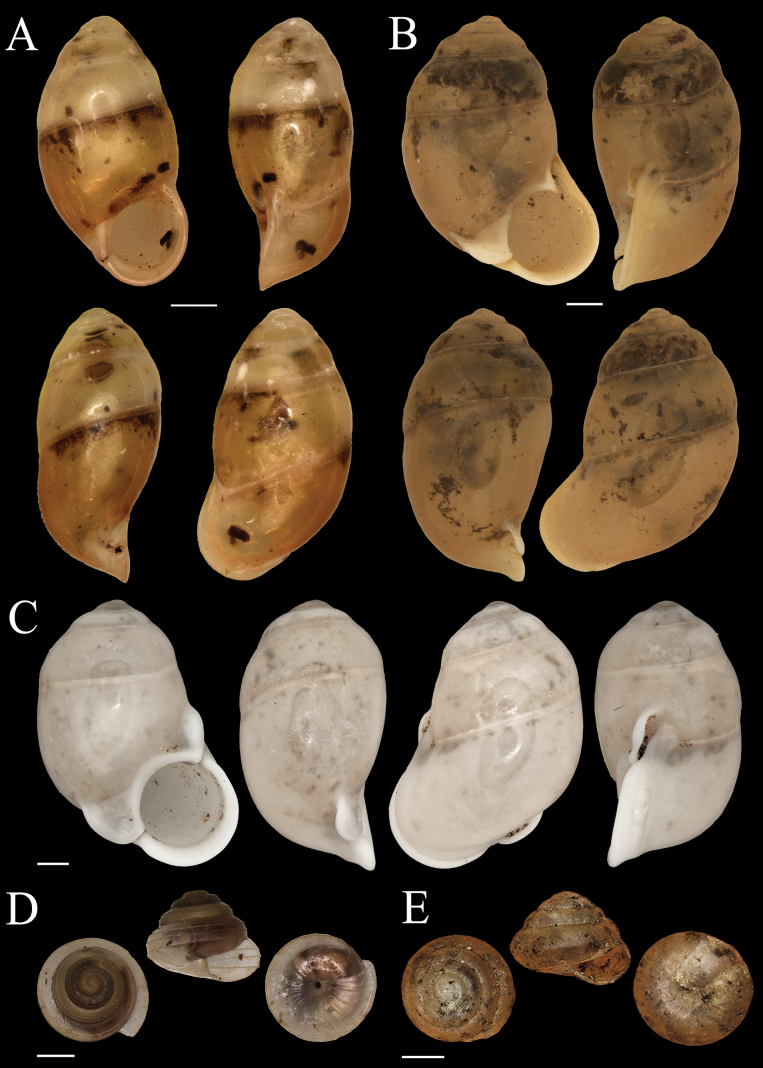
Shells of land snail species from Bacan Island **A***Moulinsiacylindrica* (Fulton, 1899) MZB Gst. 22.909 **B, C***Moulinsiasolitaria* (von Martens, 1863) MZB Gst. 22.916, 23.853 **D***Philalankakusana* (Aldrich, 1889) MZB Gst. 23.862 **E***Kaliellascandens* (Cox, 1872) MZB Gst. 23.478. Scale bars: 1 mm.

##### *Moulinsiasolitaria* (von Martens, 1863)

Fig. 5B, C

**Type locality**. Makian and Moti islands.

**Material examined.** Indonesia • North Moluccas, Bacan Is., Babang Village; 0°39.09'S, 127°32.39'E; alt. 45 m; 30 May 2022; Heryanto, N. Mujiono, I.W. Laitupa leg.; MZB Gst. 22.916/27, MZB Gst. 23.853/1, MZB Gst. 23.854/17.

**Geographic distribution and habitat.** New record for Bacan Island. The species was recorded on the islands of Makian and Moti (von Martens 1863) and Halmahera Island (Fulton 1899). In this study, the species was found in the forest edge.

**Description.** (*n* = 11) Shell moderate size with H = 8.1–9.8 mm (mean 8.8 mm); D = 5.5–6.8 mm (mean 5.9 mm); ha = 2.9–3.8 mm (mean 3.3 mm); da = 3.0–3.6 mm (mean 3.3 mm). Oval shell and slightly convex shell, yellowish, shiny, 4.5–5.5 whorls, the last whorl large and convex. Umbilicus closed, aperture highly rounded-oblique, peristome continuous. Parietal tooth pointed and receding. Sometimes a well-developed, vertically oriented tooth is present on the parietal wall, partially concealing the posterior slit that separates peristome. Parietal callus is present connecting parietal tooth and columellar tooth. Columellar tooth thickened and rectangular. Anterior and posterior canals are present.

#### ﻿Genus *Tylotoechus* Kobelt & Möllendorff, 1897

##### *Tylotoechuspfeifferianus* (H. Adams, 1869)

**Type locality.** Bacan Island.

**Geographic distribution and habitat.** Not found in this study, but this species was only recorded on Bacan Island by van Benthem Jutting (1959).

**Remarks.** The species was described by Adams (1865) in Wallace and Adams (1865) as *Pupinapfeifferi* based on the collection of Sounders from Bacan Island. In 1869, Adams corrected the species name to *Pupinapfeifferiana* because the previous name was pre-occupied by *Signepupinapfeifferi* (Dohrn, 1862). MolluscaBase (2024) has updated the name to *Tylotoechuspfeifferianus*.

#### Subclass Heterobranchia

﻿**Family Veronicellidae**

**Genus *Laevicaulis* Simroth, 1913**

##### *Laevicaulisalte* (Férussac, 1822)

**Type locality**. Pondicherry.

**Geographic distribution.** Not found in this study. This species was described as *Vaginulusalte* from Pondicherry or Puducherry, India. van Benthem Jutting (1959) recorded the species from Bacan Island.

#### ﻿Family Charopidae Hutton, 1884

**Genus *Philalanka* Godwin-Austen, 1898**

##### *Philalankakusana* (Aldrich, 1889)

Fig. 5D

**Type locality.** Southeastern Borneo.

**Material examined**. Indonesia • North Moluccas, Bacan Is., Babang Village; 0°39.09'S, 127°32.39'E; alt. 45 m; 30 May 2022; Heryanto, N. Mujiono, I.W. Laitupa leg.; MZB Gst. 23.862/1.

**Geographic distribution and habitat.** New record for Bacan Island and the first record of a Charopidae in Bacan Island. The species is widely distributed from the eastern part of Indonesia (West Papua, Moluccas) to the western part of Indonesia (Sumatra), Singapore, and Malaysia (Vermeulen and Liew 2022).

**Description.** (*n* = 1) Shell very small with H = 2.1 mm, D = 2.7 mm, ha = 1.5 mm, da = 1.9 mm, and W 4.5. The shell is whitish, conical in shape, and ornamented by two spiral ridges on the second and third whorl and three apparent spiral ridges on the body whorl.

#### ﻿Family Clausiliidae Gray, 1855

**Genus *Phaedusa* Adams & Adams, 1855**

##### *Phaedusacumingianamoluccensis* (von Martens, 1864)

Fig. 6A

**Type locality**. Halmahera Island.

**Material examined**. Indonesia • North Moluccas, Bacan Is., Sumae Village; 0°35.11'S, 127°24.19'E; alt. 54 m (M5); 1 June 2022; Heryanto, N. Mujiono, I.W. Laitupa leg.; MZB Gst. 22.933/1, MZB Gst. 23.469/2.

**Geographic distribution and habitat.** The species is distributed in Bacan Island (Loosjes 1963) and the Moluccas archipelago i.e., Halmahera Island, Ternate Island (van Benthem Jutting 1959). In this study, the species was found in karst forest areas.

**Figure 6.**
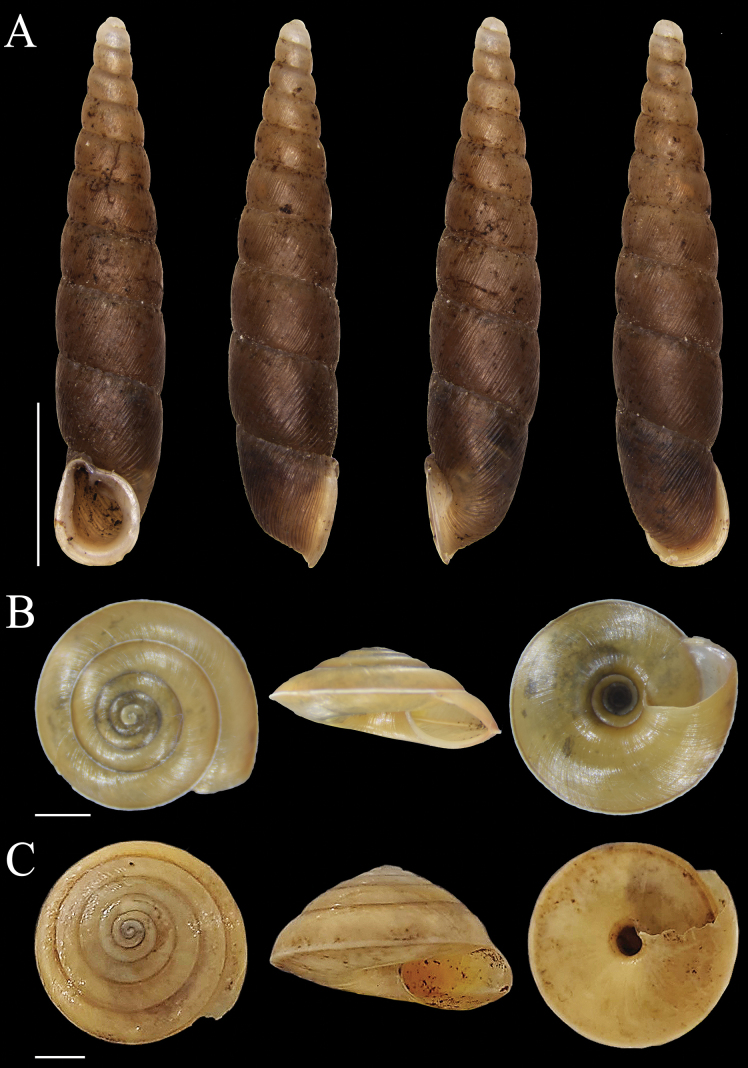
Shells of land snail species from Bacan Island **A***Phaedusacumingianamoluccensis* (von Martens, 1864) MZB Gst. 23.469 **B***Trochomorphafroggatti* (Iredale, 1941) MZB Gst. 22.198 **C***Trochomorphaternatana* (Le Guillou, 1842) MZB Gst. 22.923. Scale bars: 5 mm.

**Description.** (*n* = 2) The species was described from Djilolo, Halmahera Island as *Clausiliamoluccensis* with H = 17 mm, D = 3 mm, ha, 3 mm, da 2.3 mm (von Martens 1864). The current expedition recorded the species with shell H = 16.9–17.5 mm (mean 17.2 mm); D = 3.5–3.6 mm (mean 3.5 mm); ha = 3.3 mm; da = 2.5–2.6 mm (mean 2.5 mm); whorls 9–11 increasing in size. Hight conical shell, brownish purple. Umbilicus closed, aperture oblique, peristome continuous and has three palatal plicae.

#### ﻿Family Trochomorphidae Möllendorff, 1890

**Genus *Trochomorpha* Albers, 1850**

##### *Trochomorphafroggatti* (Iredale, 1941)

Fig. 6B

**Type locality**. Western New Guinea.

**Material examined**. Indonesia • North Moluccas, Bacan Is., Babang Village, 0°39'5.61"S, 127°32'23.17"E; alt. 45 m; 30 May 2022; Heryanto, N. Mujiono, I.W. Laitupa leg.; MZB Gst. 22.918/4; North Moluccas, Bacan Is., Sumae Village; 0°35.11'S, 127°24.19'E; alt. 54 m (M5); 1 June 2022; Heryanto, N. Mujiono, I.W. Laitupa leg.; MZB Gst. 22.932/6

**Geographic distribution and habitat.** The species is widely distributed in Indonesia. In this study, the species was found on the karst forest edge.

**Description.** (*n* = 10) Shell moderate size with H = 2.2–5.7 mm (mean 4.7 mm); D = 7.8–13.9 mm (mean 10.8 mm); ha = 1.9–4.8 mm (mean 2.8 mm); da = 2.1–5.5 mm (mean 4.0 mm); whorl 4.5–5. Conical shell, gold in colour and shiny shell, 4.5–5 Whorls increasing in size. Umbilicus open, aperture semicircle-oblique, peristome not continuous.

**Remarks.** The species was recorded on Bacan Island by Wallace (1865) as *Trochomorphaplanorbis* Lesson, 1831. However, the name *Helixplanorbis* Lesson was invalid as it was preoccupied by Linnaeus and Iredale (1941) revised the Papuan species to *Necvidena*, which was later reclassified to *Trochomorpha* (MolluscaBase 2024).

##### *Trochomorphaternatana* (Le Guillou, 1842)

Fig. 6C

**Type locality**. Ternate.

**Material examined**. Indonesia • North Moluccas, Bacan Is., Marabose Village; 0°38.97'S, 127°31.91'E; alt. 128 m (M1); 29 May 2022; Heryanto, N. Mujiono, I.W. Laitupa leg.; MZB Gst.22.910/20; North Moluccas, Bacan Is., Babang Village, 0°39.09'S, 127°32.39'E; alt. 45 m; 30 May 2022; Heryanto, N. Mujiono, I.W. Laitupa leg.; MZB Gst.22.917/22; North Moluccas, Bacan Is., Babang Village 0°37.80'S, 127°37.71'E; alt. 46 m; 31 May 2022; Heryanto, N. Mujiono, I.W. Laitupa leg.; MZB Gst.22.923/36; North Moluccas, Bacan Is., Sumae Village; 0°35.11'S, 127°24.19'E; alt. 54 m (M5); 1 June 2022; Heryanto, N. Mujiono, I.W. Laitupa leg.; MZB Gst.22.931/23.

**Geographic distribution and habitat.** The species was recorded in Bacan Island by van Benthem Jutting (1959). It was also recorded in the surrounding islands such as Ternate Is. (Le Guillou 1842) and Halmahera and Obi islands (van Benthem Jutting 1959). In this study, the species was collected in forest with karst and non-karst area, in the banana field, cocoa garden, and on the forest edge.

**Description.** (*n* = 40) Shell with moderate size H = 5.4–13.9 mm (mean 9.6 mm); D = 11.2–19.3 mm (mean 15.1 mm); ha = 3.1–6.8 mm (mean 4.9 mm); da = 3.8–8.8 mm (mean 6.9 mm); whorls 6–7 increasing in size. Conical shell, brown and shiny. Umbilicus open, aperture sickle-shaped, peristome not continuous.

**Remarks.**Pfeiffer (1861) described *Helixbatchianensis* from Bacan Island. Later in 1865, Wallace mentioned the species but classified it in the genus *Trochomorpha* and van Benthem Jutting (1959) synonymised the species with *Trochomorphaternatana*.

#### ﻿Family Chronidae Thiele, 1931

**Genus *Kaliella* W.T. Blanford, 1863**

##### *Kaliellascandens* (Cox, 1872)

Fig. 5E

**Type locality**. Port Macquarie, Australia.

**Material examined**. Indonesia • North Moluccas, Bacan Is., Babang Village 0°37.80'S, 127°37.71'E; alt. 46 m; 31 May 2022; Heryanto, N. Mujiono, I.W. Laitupa leg.; MZB Gst. 23.478/1.

**Geographic distribution and habitat.** New record for Bacan Island. In the Moluccas, the species was known from Ema, South Ambon, and other larger surrounding islands such as Hitulama, North Ambon, and Sirisori in Saparua (Boettger 1891). The species is widely distributed in Indonesia. In this study, the species was found in karst forest.

**Description.** (*n* = 1) Shell small size with H = 2.2 mm; D = 2.8 mm; ha = 0.5 mm; da = 0.8 mm; whorl 4. Hight conical shell, brown transparent shell. Umbilicus closed, aperture sickle-shaped, peristome not continuous.

**Remarks.**Boettger (1891) described this species as *Kaliellaindifferens* from Ema, South Ambon. Based on Vermeulen et al (2015) the species name was synonymised to *Kaliellascandens*.

#### ﻿Family Microcystidae Thiele, 1931

**Genus *Lamprocystis* Pfeiffer, 1883**

##### *Lamprocystisambonica* Boettger, 1891

Fig. 7A

**Type locality**. Ema, Ambon.

**Material examined.** Indonesia • North Moluccas, Bacan Is., Babang Village 0°37.80'S, 127°37.71'E; alt. 46 m; 31 May 2022; Heryanto, N. Mujiono, I.W. Laitupa leg.; MZB Gst. 23.476/ 8.

**Geographic distribution and habitat.** The species was recorded in Bacan Is. (Boettger 1891). It was also recorded from the surrounding islands such as Ema (Ambon), Banda Neira, and Haruku Island (Boettger 1891), and Halmahera Island (van Benthem Jutting 1959). In this study, the species was found in karst forest.

**Figure 7.**
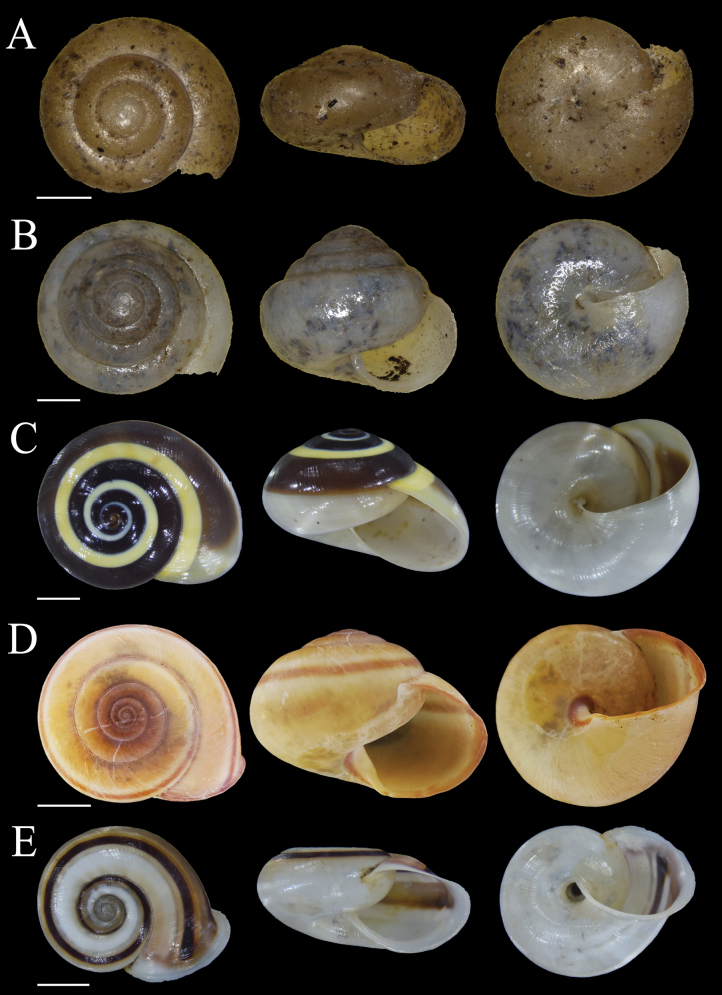
Shells of land snail species from Bacan Island **A***Lamprocystisambonica* Boettger, 1891 MZB Gst. 23.476 **B***Lamprocystis* ‘Bacan 1’ MZB Gst.23.474 **C***Xestacitrina* (Linnaeus, 1758) MZB Gst. 22.939 **D***Cochlostylapubicepa* von Martens, 1864 MZB Gst. 22.907 **E***Cristigibbaexpansa* (Pfeiffer, 1861) MZB Gst. 22.937. Scale bars: 1 mm (**A, B**); 5 mm (**C, E**); 10 mm (**D**).

**Description.** (*n* = 8) Shell small size with H = 2.3–2.8 mm (mean 2.5 mm); D = 3.7–4.4 mm (mean 4.2 mm); ha = 1.5–1.9 mm (mean 1.7 mm); da = 1.2–1.9 mm (mean 1.5 mm). Shell conical, yellowish golden in colour, shiny, transparent; 4–4.5 whorls increasing in size. Umbilicus closed, aperture sickle-shaped, peristome not continuous.

##### *Lamprocystis* ‘Bacan 1’

Fig. 7B

**Material examined.** Indonesia • North Moluccas, Bacan Is., Babang Village 0°37.80'S, 127°37.71'E; alt. 46 m; 31 May 2022; Heryanto, N. Mujiono, I.W. Laitupa leg.; MZB Gst.23.474/2, MZB Gst.23.477/3; North Moluccas, Bacan Is., Sumae Village; 0°35.11'S, 127°24.19'E; alt. 54 m (M5); 1 June 2022; Heryanto, N. Mujiono, I.W. Laitupa leg.; MZB Gst.23.475/11.

**Geographic distribution and habitat.** The species was recorded in karst forest.

**Description.** (*n* = 16) Shell small size with H = 3.3–5.3 mm (mean 4.3 mm); D = 3.9–7.1 mm (mean 5.3 mm); ha = 1.1–2.5 mm (mean 1.5 mm); da = 1.6–2.7 mm (mean 2.2 mm). High conical shell, shiny shell with white colour and transparent, 4.5–5 whorls increasing in size and convex. Umbilicus close, aperture sickle-shaped, peristome not continuous.

**Remarks.** The species is similar to *Lamprocystissubangulata* Boettger, 1891 but differs in having a more convex whorl and rounded penultimate whorl. The species *L.subangulata* was recorded in Ema (South Moluccas) and in Carbau Mountain and Waai (Boettger 1891).

#### ﻿Family Ariophantidae Godwin-Austen, 1888

##### *Naninaignescens* (Pfeiffer, 1861)

**Type locality.** Bacan Island.

**Remarks.** Not found in this study. This species was described from Bacan Island as *Helixignescens* and figured (Pfeiffer 1861: pl. 2, fig. 1).

##### *Naninaluctuosa* Beck, 1837

**Type locality.** Indo-Australia archipelago.

**Remarks.** Not found in this study. This species was described by Beck (1937) and figured by Tryon (1886: pl. 19, fig. 85).

##### *Naninasulfurata* von Martens, 1864

**Type locality.** Bacan and Halmahera islands.

**Remarks.** Not found in this study. This species was described from Bacan and Halmahera islands and figured in von Martens (1867: pl. 8, figs 1, 1b).

#### ﻿Genus *Xesta* Albers, 1850

##### *Xestacitrina* (Linnaeus, 1758)

Fig. 7C

**Type locality.** Jamaica.

**Material examined**. Indonesia • North Moluccas, Bacan Is., Sumae Village; 0°35.11'S, 127°24.19'E; alt. 54 m (M5); 1 June 2022; Heryanto, N. Mujiono, I.W. Laitupa leg.; MZB Gst.22.939/6.

**Geographic distribution and habitat.** New record for Bacan Island. The species was recorded in the Moluccan region including Ambon, Seram, and Buru islands (von Martens 1867), and Halmahera and Ternate islands (van Benthem Jutting 1959). The species can also be found in the Cendrawasih Bay on Biak Island (Tapparone-Canefri 1886). In this study, the species was found in karst forest.

**Description.** (*n* = 5) Shell large with H = 16.3–17.0 mm (mean 16.7 mm); D = 23.3–25.8 mm (mean 24.4 mm); ha = 10.0–12.6 mm (mean 10.7 mm); da = 11.4–14.9 mm (mean 12.6 mm). Rounded shell, dominant white colour with brown and yellow stripes from apex until body whorl, 4–5 whorls increasing in size. Umbilicus closed, aperture semicircle-oblique, peristome not continuous.

**Remarks.** The species was described by Linnaeus (1758) in *Helix*. The shell of *X.citrina*, *N.ignescens*, *N.luctuosa*, and *N.sulfurata* are very similar. Systematic revision with integrative approaches (phylogenetic, morphology, and anatomy) of this group is necessary to assess whether the colour patterns are useful for species diagnosis, as well as to clarify their taxonomy.

#### ﻿Family Camaenidae Pilsbry, 1895

**Genus *Cochlostyla* A. Férussac, 1821**

##### *Cochlostylapubicepa* von Martens, 1864

Fig. 7D

**Type locality**. Halmahera and Bacan islands.

**Material examined.** Indonesia • North Moluccas, Bacan Is., Sumae Village; 0°35.11'S, 127°24.19'E; alt. 54 m (M5); 1 June 2022; Heryanto, N. Mujiono, I.W. Laitupa leg.; MZB Gst. 22.934/9; North Moluccas, Bacan Is., Marabose Village; 0°38.97'S, 127°31.91'E; alt. 128 m (M1); 29 May 2022; Heryanto, N. Mujiono, I.W. Laitupa leg.; MZB Gst. 22.907/4.

**Geographic distribution and habitat.** The species was recorded in Halmahera and Bacan islands (von Martens 1864), as well as on Obi Island (van Benthem Jutting 1959). In this study, the species was collected in the banana field and karst forest.

**Description.** (*n* = 11) Shell large with H = 14.6–24.0 mm (mean 18.5 mm); D = 17.3–31.8 mm (mean 23.0 mm); ha = 10.2–16.9 mm (mean 13.8 mm); da = 8.8–15.0 mm (mean 11.6 mm); whorl 5–5.5. Shell yellowish brown with dark brown spiral band on the body whorl.

#### ﻿Genus *Cristigibba* Tapparone Canefri, 1883

##### *Cristigibbacorniculum* (Hombron & Jacquinot, 1847)

**Type locality.** New Guinea.

**Remarks.** Not found in this study. This species was originally described in *Helix*. It was recorded from Bacan Island by Wallace and Adams (1865).

##### *Cristigibbaexpansa* (Pfeiffer, 1861)

Fig. 7E

**Type locality**. Bacan Island.

**Material examined.** Indonesia • North Moluccas, Bacan Is., Sumae Village; 0°35.11'S, 127°24.19'E; alt. 54 m (M5); 1 June 2022; Heryanto, N. Mujiono, I.W. Laitupa leg.; MZB Gst. 22.937/29.

**Geographic distribution and habitat.** The species is so far only recorded in Bacan Island, in karst forest.

**Description.** (*n* = 5) The species was described by Pfeiffer (1861) as *Helix* with H = 10 mm and D = 17.5–22 mm. The current expedition recorded shells with smaller sizes: H = 6.7–10.4 mm (mean 8.8 mm), D = 10.1–18.8 mm (mean 13.8 mm), ha = 5.4–8.0 mm (mean 6.8 mm), da = 4.5–7.8 mm (mean 6.2 mm) and whorl 4–4.5.

#### ﻿Genus *Landouria* Godwin-Austen, 1918

##### *Landouriawinteriana* (Pfeiffer, 1842)

**Type locality.** Java.

**Remarks.** Not found in this study. Based on the recent systematic revision of *Landouria* in Java, the species of *L.winteriana* may have distributed in Java and Sumatera islands of Indonesia but the dispersal to the eastern part of Indonesia is unlikely (Nurinsiyah et al. 2019). The species in *Landouria* are often misidentified due to the similarities of shell morphology. Thus, the species in Bacan recorded by Boettger (1891) as Helix (Plectotropis) winteriana and Smith (1896) as Eulota (Plectotropis) winteriana might belong to different species; further collections are necessary.

#### ﻿Genus *Obba* H. Beck, 1837

##### *Obbasubgranulata* Sykes, 1904

**Type locality**. Bacan Island.

**Remarks.** Not found in this study. This species was described from Bacan Island as *Obbasubgranulata* and figured by Sykes (1904: pl. 9, figs 5, 6).

#### ﻿Genus *Papuina* E. von Martens, 1860

##### *Papuinanodifera* (Pfeiffer, 1861)

Fig. 8A

**Type locality**. Bacan Island.

**Material examined**. Indonesia • North Moluccas, Bacan Is., Sawadai Village; 0°45.18'S, 127°27.12'E; alt. 84 m; 29 May 2022; Heryanto, N. Mujiono, I.W. Laitupa leg.; MZB Gst. 24.187/1.

**Geographic distribution and habitat.** So far, this species has only been found on Bacan Island (van Benthem Jutting 1959). In this study, the species was found in the cocoa garden.

**Remarks.** The species was described by Pfeiffer (1861) from the collection of Alfred Russel Wallace as *Helixnodifera* with the size H = 18 mm and D = 24–30 mm. The current expedition collected only one specimen of *P.nodifera* with a smaller shell, H = 14.3 mm, D = 18.3 mm, ha = 6.5 mm, da = 11.9 mm, and with 4.5 whorls.

**Figure 8.**
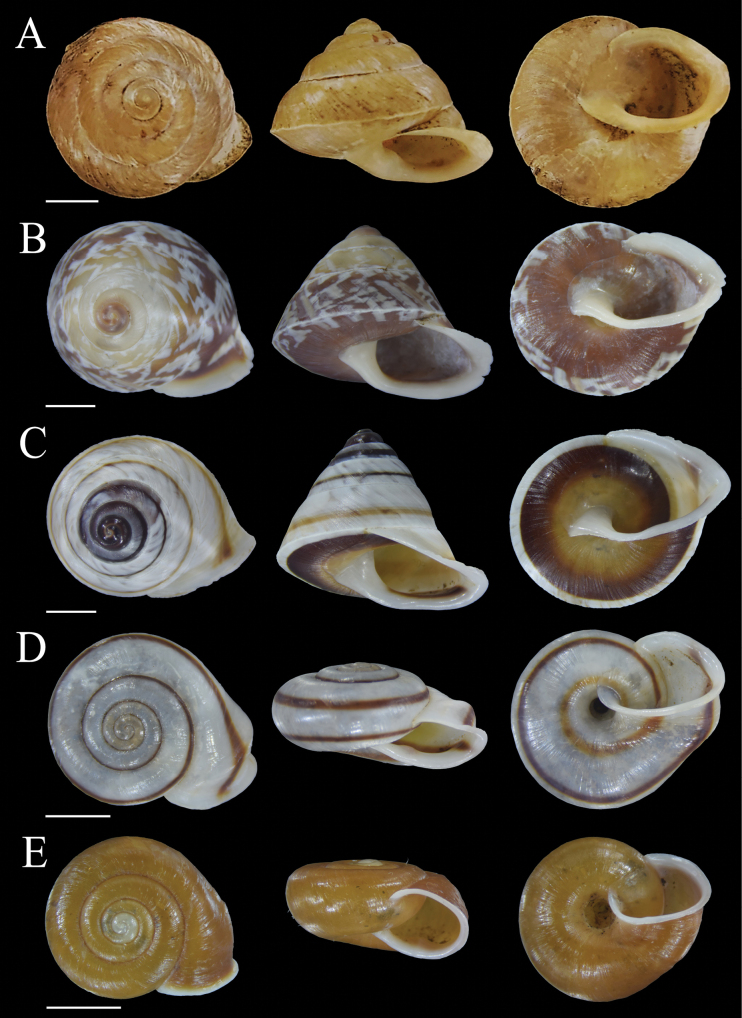
Shells of land snail species from Bacan Island **A***Papuinanodifera* (Pfeiffer, 1861) MZB Gst. 24.187 **B***Papuinapileolus* (Férussac, 1821) MZB Gst. 22.921 **C***Papuinarhynchostoma* (Pfeiffer, 1861) MZB Gst. 22.938 **D***Planispiraquadrifasciata* (Le Guillou, 1842) MZB Gst. 23.944 **E***Vulnusendoptycha* (von Martens, 1864) MZB Gst. 22.920. Scale bars: 5 mm.

##### *Papuinaohlendorfii* Kobelt, 1897

**Type locality**. Bacan.

**Remarks.** Not found in this study. It was recorded in Bacan Island and described and illustrated by Kobelt (1897: pl. 8, figs 6, 7).

##### *Papuinapileolus* (Férussac, 1821)

Fig. 8B

**Type locality.** Unknown.

**Material examined**. Indonesia • North Moluccas, Bacan Is., Babang Village 0°37.80'S, 127°37.71'E; alt. 46 m (M4); 31 May 2022; Heryanto, N. Mujiono, I.W. Laitupa leg.; MZB Gst. 22.921/4; North Moluccas, Bacan Is., Marabose Village; 0°38.97'S, 127°31.91'E; alt. 128 m (M1); 29 May 2022; Heryanto, N. Mujiono, I.W. Laitupa leg.; MZB Gst. 22.908/3.

**Geographic distribution and habitat.** The species was recorded in southern Halmahera, Bacan Island, and near Telaga Manga Joang (van Benthem Jutting 1959). In this study, the species was found in the banana plantation in a non-karst area and in the karst forest.

**Description.** (*n* = 7) The species was described by Férussac (1821) as *Helixpileolus*. Shell large with: H = 17.1–20.5 mm (mean 18.6 mm); D = 22.0–25.9 mm (mean 23.9 mm); ha = 8.0–9.8 mm (mean 8.6 mm); da = 8.1–11.5 mm (mean 10.1 mm). High conical shell, yellowish brown colour with white abstract pattern, 5–5.5 whorls increasing in size. Umbilicus closed, aperture oblique outer lips slightly thickened, peristome not continuous.

##### *Papuinarhynchostoma* (Pfeiffer, 1861)

Fig. 8C

**Type locality**. Bacan Island.

**Material examined.** Indonesia • North Moluccas, Bacan Is., Sumae Village; 0°35.11'S, 127°24.19'E; alt. 54 m (M5); 1 June 2022; Heryanto, N. Mujiono, I.W. Laitupa leg.; MZB Gst. 22.938/17.

**Geographic distribution and habitat.** So far, this species has only been found on Bacan Island (van Benthem Jutting 1959). In this study, the species was found in the karst forest.

**Remarks.** (*n* = 5) Shell large with H = 17.3–20.8 mm (mean 19.7 mm); D = 19.9–24.8 mm (mean 23.2 mm); ha = 7.0–10.0 mm (mean 8.6 mm); da = 9.2–14.2 mm (mean 10.8 mm). High conical shell, brownish white with yellowish strips, 5–5.5 rounded whorls increasing in size. Umbilicus closed, aperture oblique, and outer lips slightly thickened, peristome not continuous.

##### *Papuinavitrea* (Férussac, 1821)

**Type locality.** Unknown.

**Remarks.** Not found in this study. This species was described as *Helixvitrea*. Tapparone Canefri (1883) recorded the species from Bacan Island.

#### ﻿Genus *Planispira* H. Beck, 1837

##### *Planispiraatrofusca* (Pfeiffer, 1861)

**Type locality**. Bacan Island.

**Remarks.** Not found in this study. This species was described from Bacan Island as *Helixatrofusca* and figured in Pfieffer (1861: pl. 3, fig. 3).

##### *Planispirabiconvexa* (von Martens, 1864)

**Type locality**. Little Tawalli Island.

**Remarks.** Not found in this study. This species was described from Tawali Kecil Island near Bacan Island as *Helixbiconvexa*. The species was figured later in von Martens (1867: pl. 16, fig. 13).

##### *Planispiraexceptiuncula* (Férussac, 1823)

**Type locality.** Australia.

**Remarks.** Not found in this study. This species was described from Australia *Helixexceptiuncula* and figured in Pilsbry (1890: pl. 45, figs 50–53; pl. 65, figs 84–87). van Benthem Jutting (1959) recorded the species from Bacan Island.

##### *Planispirakurri* (Pfeiffer, 1848)

**Type locality.** Unknown.

**Remarks.** Not found in this study. This species was described as *Helixkurri*. It was recorded from Bacan Island by Wallace and Adams (1865) and figured by Pilsbry (1890: pl. 45, figs 21–23).

##### *Planispiralacteocincta* Smith, 1896

**Type locality**. Bacan Island.

**Remarks.** Not found in this study. This species was described from Bacan Island as Planispira (Cristigibba) lacteocincta and figured by Smith (1896: figs 3, 4).

##### *Planispirraloxotropis* (Pfeiffer, 1850)

**Type locality.** Moluccas.

**Remarks.** Not found in this study. This species was described as *Helixloxotropis* and figured by Pilsbry (1890: pl. 46, figs 60–64, 68). van Benthem Jutting (1959) recorded the species from Bacan Island.

##### *Planispiraquadrifasciata* (Le Guillou, 1842)

Fig. 8D

**Type locality**. Ternate.

**Material examined.** Indonesia Indonesia • North Moluccas, Bacan Is., Sumae Village; 0°35.11'S, 127°24.19'E; alt. 54 m (M5); 1 June 2022; Heryanto, N. Mujiono, I.W. Laitupa leg.; MZB Gst. 23.944/2.

**Geographic distribution and habitat.** New record for Bacan Island. The species was recorded in Ternate (Le Guillou 1842), Halmahera, and Obi islands (van Benthem Jutting 1959). In this study, the species was found in karst forest.

**Description.** (*n* = 5) Shell large with H = 7.4–9.8 mm (mean 8.8 mm); D = 11.9–19.6 mm (mean 16.2 mm); ha = 6.0–8.6 mm (mean 6.9 mm); da = 4.7–7.0 mm (mean 6.3 mm). Rounded shell, white base colour with brown strip, 4–4.5 whorl increasing in size, last whorl has a wave that is close to the aperture. Umbilicus open, aperture oblique, peristome not continuous.

##### *Planispirathetis* (Pfeiffer, 1851)

**Type locality.** Unknown.

**Remarks.** Not found in this study. This species was described as *Helixthetis* and figured by Pilsbry (1890: pl. 56, figs 74–76). Van Benthem Jutting (1959) recorded the species from Bacan Island.

##### *Planispirazonalis* (Férussac, 1821)

**Type locality.** Moluccas.

**Remarks.** Not found in this study. This species was described *Helixzonalis* from Moluccan islands without mentioning a specific island or place. The species was figured by Pilsbry (1890: pl. 45, figs 24, 25, 29, 30). Van Benthem Jutting (1959) recorded the species from Bacan Island.

##### *Planispirazonaria* (Linnaeus, 1767)

**Type locality.** Southern Europe.

**Remarks.** Not found in this study. This species was described as *Helixzonaria*. von Martens (1867: pl. 16, figs 6–11; pl. 19, fig. 6) recorded the species from the Moluccan islands such as Ambon, Seram, Buru and Banda islands. Van Benthem Jutting (1959) recorded the species from Bacan Island.

#### ﻿Genus *Pseudopapuina* F. Haas, 1934

##### *Pseudopapuinascheepmakeri* (Pfeiffer, 1850)

**Type locality.** Moluccas.

**Remarks.** Not found in this study. This species was described as *Helixscheepmakeri* and figured by Pilsbry (1890: pl. 55, figs 40, 48, 49). van Benthem Jutting (1959) recorded the species from Bacan Island. Based on the figures from Pilsbry’s plate, this species is very similar to *P.biconvexa* and they are possibly synonymous.

#### ﻿Genus *Pyrochilus* Pilsbry, 1893

##### *Pyrochiluspyrostoma* (Férussac, 1821)

**Type locality**. East Indies.

**Remarks.** Not found in this study. This species was described as *Helixpyrostoma* and figured by Pilsbry (1890: pl. 20, fig. 42). van Benthem Jutting (1959) recorded the species from Bacan Island.

##### *Pyrochilussulcocinctus* (von Martens, 1864)

**Type locality.** Halmahera.

**Remarks.** Not found in this study. This species was described as *Cochlastylasulcocincta* and figured by Pilsbry (1890: pl. 59, figs 39–41) as *Helixsulcocincta*. van Benthem Jutting (1959) reassigned it to the genus *Pyrochilus* and recorded the species from Bacan Island.

##### *Pyrochilusxanthostoma* (von Martens, 1867)

**Type locality**. Bacan Island.

**Remarks.** Not found in this study. This species was described as *Helixxanthostoma* Herklots; however, we cannot find any further description. The species described by von Martens (1867) was from Bacan Island.

#### ﻿Genus *Sulcobasis* Tapparone Canefri, 1883

##### *Sulcobasisconcisarubra* (Albers, 1857)

**Type locality.** Misool.

**Remarks.** Not found in this study. This species was described as *Helixrubra* from Aru Island. However, based on the examination of Boettger (1914), the species which Albers (1857) described referred to other subspecies *Sulcobasisconcisacumingi* Gude, 1906. True species of *Sulcobasisconcisarubra* were the ones recorded from the Moluccas and Bacan Island (Boettger 1914).

#### ﻿Genus *Vulnus* Sykes, 1904

##### *Vulnusendoptycha* (von Martens, 1864)

Fig. 8E

**Type locality**. Ternate and Bacan islands.

**Material examined**. INDONESIA • North Moluccas, Bacan Is., Babang Village, 0°39’5.61’’S, 127°32’23.17’’E; alt. 45 m; 30 May 2022; Heryanto, N. Mujiono, I.W. Laitupa leg.; MZB Gst. 22.919/3; North Moluccas, Bacan Is., Babang Village 0°37.80'S, 127°37.71'E; alt. 46 m; 31 May 2022; HER, NM, ILW leg.; MZB Gst. 22.920/20; North Moluccas, Bacan Is., Sumae Village; 0°35.11'S, 127°24.19'E; alt. 54 m (M5); 1 June 2022; Heryanto, N. Mujiono, I.W. Laitupa leg.; MZB Gst. 22.936/2.

**Geographic distribution and habitat.** The species was recorded in Bacan Is. and Ternate Is. (von Martens 1864). In this study, the species was collected in the banana field and karst forest.

**Description.** (*n* = 14) Shell moderate in size with H = 5.3–7.9 mm (mean 6.8); D = 11.0–14.9 mm (mean 13.2 mm); ha = 4.0–5.9 mm (mean 5.2 mm); da = 2.9–3.9 mm (mean 3.5 mm). Flat shell, brown and shiny, 3.5–4 whorls increasing in size, last whorl rounded and large. Presence of a basal tooth, and a groove perpendicular to the coiling axis at the periphery of the body whorl ~ 1/4 of a whorl before the peristomal thickening. Umbilicus open, aperture oblique, peristome not continuous.

## Supplementary Material

XML Treatment for Diancta batubacan

## ﻿Additional information

### Author ORCIDs

Ayu Savitri Nurinsiyah https://orcid.org/0000-0003-2595-6482

Ahmad Ghifari Prasetia https://orcid.org/0009-0002-2973-9741

Nova Mujiono https://orcid.org/0000-0002-5792-180X

Heryanto https://orcid.org/0009-0009-3175-9726

### Data availability

All of the data that support the findings of this study are available in the main text.

## References

[B1] Albers von (1857) Diagnosen neuer Heliceen mit gelegentlicher berichtigung einiger älteren arten. Malakozoologische Blätter 18: 1–232.

[B2] Aldrich TH (1889) Notes upon a collection of shells from Borneo with descriptions of new species. Journal of the Cincinnati Society of Natural History 12: 23–26 [pl. 3].

[B3] Badan Pusat Statistik Kabupaten Halmahera Selatan (2023) Statistik Daerah Kabupaten Halmahera Selatan 2023. BPS Kabupaten, Halmahera Selatan, 73 pp.

[B4] Beck H (1837) Index Molluscorum præsentis ævi musei principis authustissimi Christiani Frederici, Copenhagen, 124 pp. 10.5962/bhl.title.77331

[B5] Boettger O (1891) Ad. Strubell´s Konchylien aus Java II und von den Molukken. Bericht über die Senckenbergische Naturforschende Gesellschaft in Frankfurt am Main 1891: 241–318 [pls 3, 4].

[B6] Boettger CR (1914) On Sulcobasisconcisa (Fér.) and its nearest allies. Proceedings of the Malacological Society of London 9(2): 181–188. 10.1093/oxfordjournals.mollus.a063561

[B7] Boonmachai T Bergey EA Wongsawad C Nantarat N (2024) Influence of limestone and anthropogenic activities on land snail communities in Satun Province, Thailand. Science of the Total Environment 912: 169372. 10.1016/j.scitotenv.2023.169372 38104843

[B8] Caldwell RS (1993) Macroinvertebrates and their relationship to coarse woody debris: with special reference to land snails. Proceedings of the Workshop on Coarse Woody Debris in Southern Forests: Effects on Biodiversity, 49–54.

[B9] Cox J (1872) Description of a new Volute and twelve new species of land-shells from Australia and the Solomon Islands. Proceedings of the Zoological Society of London 1871: 643–647.

[B10] Dohrn H (1862) Description of new operculated land shells. Proceedings of the Zoological Society of London 30(1): 181–186. 10.1111/j.1469-7998.1862.tb06493.x

[B11] Douglas DD Brown DR Pederson N (2013) Land snail diversity can reflect degrees of anthropogenic disturbance. Ecosphere 4(2): 1–14. 10.1890/ES12-00361.1

[B12] Férussac AEPF d’Audebard de (1819–1832) Histoire naturelle générale et particulière des mollusques terrestres et fluviatiles tant des espèces que l’on trouve aujourd’hui vivantes, que des dépouilles fossiles de celles qui n’existent plus; classés d’après les caractères essentiels que présentent ces animaux et leurs coquilles. J.-B. Bailliere, Paris, 402 pp. 10.5962/bhl.title.124603

[B13] Fulton H (1899) A list of the species of land Mollusca collected by Mr. W. Doherty in the Malay Archipelago; with descriptions of some supposed new species and varieties. Proceedings of the Malacological Society of London 3(1898–1899): 212–219 [pl. 11].

[B14] Godwin-Austen HH (1898) On Philalanka, a new subgenus of Endodonta, with descriptions of two new species from the Indian region. Proceedings of the Zoological Society of London 1897(65): 489–496.

[B15] Grateloup JPS (1840) Mémoire descriptif sur plusieurs espèces de coquilles nouvelles ou peu connues de mollusques exotiques vivants, terrestres, fluviatiles et marins. Actes de La Sociéte Linnéenne de Bordeaux 17: 394–455. 10.5962/bhl.title.14738

[B16] Greķe K (2012) Non-marine Mollusca of Gebe Island, North Moluccas. Vernate 31: 225–240.

[B17] Greķe K (2017) Taxonomic Review of Diplommatinidae (Caenogastropoda: Cyclophoroidea) from Wallacea and the Papuan Region. Biodiversity, Biogeography and Nature Conservation in Wallacea and New Guinea 3: 151–316.

[B18] Grimpe G Hoffmann H (1925) Die Nacktschnecken von Neu-Caledonien, den Loyalty-Inseln und den Neuen-Hebriden. Zoologie 3(3): 339–476.

[B19] Gude GK (1904) A classified list of the Helicoid land shells of Asia Part VII. Journal of Malacology 11(1): 83–98.

[B20] Hausdorf B (2018) Beyond Wallace’s line – dispersal of Oriental and Australo-Papuan Land-Snails across the Indo-Australian Archipelago. Zoological Journal of the Linnean Society 185: 66–76. 10.1093/zoolinnean/zly031

[B21] Heryanto Heryanto Mujiono N Laitupa IW Nurinsiyah AS (2023) Two species of Diplommatina and a new species of Palaina (Gastropoda: Cyclophoroidea: Diplommatinidae) from Moti Island, North Moluccas, Indonesia. Raffles Bulletin of Zoology 71: 331–336. 10.26107/RBZ-2023-0025

[B22] Heryanto (2012) Keanekaragaman Keong Darat di Dua Macam Habitat Makro di Gunung Slamet Jawa Tengah in Ekologi Gunung Slamet. LIPI Press, Indonesia, 193–204.

[B23] Hombron JB Jacquinot H (1847) Atlas d’Histoire Naturelle. Zoologie par MM. Hombron et Jacquinot, chirurgiens de l’expédition. in: Voyage au pole sud et dans l’Océanie sur les corvettes l’Astrolabe et la Zélée pendant les années 1837-1838-1839-1840 sous le commandement de M. Dumont D’Urville capitaine de vaisseau publié sous les auspices du département de la marine et sous la direction supérieure de M. Jacquinot, capitaine de Vaisseau, commandant de La Zélée. Vingt-troisieme livraison. Mollusques [pls 4, 5, 6, 8, 10].

[B24] Iredale T (1941) A basic list of the land Mollusca of Papua. The Australian Zoologist 10(1): 51–94.

[B25] Kobelt W (1897) Land- Und Süsswasserkonchylien. Abhandlungen Der Senckenbergischen Naturforschenden Gesellschaft 24(1): 17–92.

[B26] Kobelt W (1902) Das Tierreich. Eine Zusammenstellung und Kennzeichnung der rezenten Tierformen. 16. Lieferung. Mollusca. Cyclophoridae. Das Tierreich. [XXXIX +] 662 pp. [1 map]

[B27] Le Guillou E (1842) Description de vingt-sept espèces d’hélices nouvelles. Revue Zoologique 5: 136–141.

[B28] Lesson RP (1831) Chapitre XI. Mollusques, Annélides et Vers; par R.-P. Lesson, 239–456. In Lesson RP (1830–1831) Voyage autour du monde, Exécuté par Ordre du Roi, sur La Corvette de Sa Majesté, La Coquille, pendant les années 1822, 1823, 1824 et 1825, sous le ministère et conformément aux Instructions de S.E.M. le Marquis de Clermont-Tonnerre, Ministre de la Marine; Et publié sous les auspices de son Excellence Mgr le Cte de Chabrol, Ministre de la Marine et des Colonies, par M.L.I. Duperrey, Capitaine de Frégate, Chevalier de Saint-Louis et Membre de la Légion d’Honneur, Commandant de l’Expédition. Zoologie, par M. Lesson. Tome Second. 1^ere^ Partie. Arthus Bertrand, Imprimerie de Firmin Didot, Paris, 471 pp.

[B29] Linnaeus C (1758) Systema Naturae per regna tria naturae, secundum classes, ordines, genera, species, cum characteribus, differentiis, synonymis, locis. Tomus I. Impensis Direct, Laurentii Salvii, Stockholm, 500 pp. 10.5962/bhl.title.542

[B30] Linnaeus C (1767) Systema naturae per regna tria naturae: secundum classes, ordines, genera, species, cum characteribus, differentiis, synonymis, locis. Tomus I, Pars II. Impensis Direct, Laurentii Salvii, Stockholm, 1032 pp. 10.5962/bhl.title.158187

[B31] Loosjes FE (1963) Supplement to a monograph of the Indo-Australian Clausiliidae. Zoologische Mededelingen 38(9): 153–169.

[B32] Malaihollo JFA Hall R (1996) The geology and tectonic evolution of the Bacan region. In Hall, R and Blundell, D (eds). Tectonic Evolution of Southeast Asia. Geological Society Special Publication 106: 483–197. 10.1144/GSL.SP.1996.106.01.30

[B33] Möllendorff OF (1902) Binnenmollusken aus Niederländisch Indien. Nachrichtsblatt der deutschen malakozoologischen Gesellschaft 34(11/12): 185–207.

[B34] MolluscaBase [Eds] (2024) MolluscaBase. *Trochomorphafroggatti* (Iredale, 1941). https://www.molluscabase.org/aphia.php?p=taxdetails&id=1345820 [accessed 5 Mar 2025]

[B35] Neubert E Bouchet P (2015) The Diplommatinidae of Fiji – a hotspot of Pacific land snail biodiversity (Caenogastropoda, Cyclophoroidea). ZooKeys 487: 1–85. 10.3897/zookeys.487.8463 PMC4366685 25829849

[B36] Nurhayati PA Affandi M Nurinsiyah AS (2021) Diversity and abundance of terrestrial Gastropods on the slopes of Mount Arjuna-Welirang, East Java, Indonesia. Biodiversitas 22(10): 4193–4102. 10.13057/biodiv/d221009

[B37] Nurinsiyah AS Fauzia H Hennig C Hausdorf B (2016) Native and introduced land snail species as ecological indicators in different land use types in Java. Ecological Indicators 70: 557–565. 10.1016/j.ecolind.2016.05.013

[B38] Nurinsiyah AS Neiber MT Hausdorf B (2019) Revision of the land snail genus Landouria Godwin-Austen, 1918 (Gastropoda, Camaenidae) from Java. European Journal of Taxonomy 526: 1–73. 10.5852/ejt.2019.526

[B39] Pearce TA (2008) When a snail dies in the forest, how long will the shell persist. Effect of dissolution and micro-bioerosion. American Malacological Bulletin 26: 111–117. 10.4003/006.026.0211

[B40] Pfeiffer L (1842) Symbolae ad historiam Heliceorum, Section Altera. Theodor Fischer, Kassel, 147 pp. 10.5962/bhl.title.11903

[B41] Pfeiffer L (1851) Descriptions of forty-three new species of Cyclostomacea, from the collection of H. Cuming, Esq. Proceedings of the Zoological Society of London 19: 242–251. 10.1111/j.1096-3642.1851.tb01173.x

[B42] Pfeiffer L (1861) Descriptions of forty-seven new species of land-shells, from the collection of H. Cuming, Esq. Proceedings of the Zoological Society of London 29(2): 20–29.

[B43] Phung CS Heng PS Liew TS (2017) Land snails of *Leptopoma* Pfeiffer, 1847 in Sabah, Northern Borneo (Caenogastropoda: Cyclophoridae): an analysis of molecular phylogeny and geographical variations in shell form. PeerJ 5(4): 1–18. 10.7717/peerj.3981 PMC5669252 29104827

[B44] Phung CS Choo MH Liew TS (2022) Sexual dimorphism in shell size of the land snail Leptopoma perlucidum (Caenogastropoda: Cyclophoridae). PeerJ 10(4): 1–12. 10.7717/peerj.13501 PMC9150688 35651743

[B45] Pilsbry HA (1890) Manual of Conchology, structural and systematic with illustrations of the species, Vol. VI, Helicidae Vol. IV. Binder and Kelly Philadelphia, 324 pp [pl. 69].

[B46] Preston HB (1913) New minute terrestrial and aquatic Mollusca from the Dutch East Indian Island of Beilan-Beilan, with descriptions of four new genera and subgenera. The Annals and magazine of natural history 12(71): 432–443. 10.1080/00222931308693421

[B47] Raheem DC Naggs F Preece RC Mapatuna Y Kariyawasam L Eggleton P (2008) Structure and conservation of Sri Lankan land-snail assemblages in fragmented lowland rainforest and village home gardens. Journal of Applied Ecology 45: 1019–1028. 10.1111/j.1365-2664.2008.01470.x

[B48] Rosales R Lillo E Alcazar SM Colita L Caballero J Malaki AB (2020) Species composition, relative abundance, and distribution of land snail species in Mt. Lantoy Key Biodiversity Area, Cebu, Philippines. Biodiversitas Journal of Biological Diversity 21(11): 5438–5447. 10.13057/biodiv/d211152

[B49] Rowson B Wood H (2015) Shells from Alfred Russel Wallace in the National Museum of Wales. Mollusc World 37: 19–23.

[B50] Schepman MM (1919) On a collection of land- and freshwater Mollusca and a few marine Mollusca, chiefly collected by DR. H.A. Lorentz from New- Guinea, The Aru Islands, Timor and Borneo. Nova Guinea. Résultats de l’expédition scientifique néerlandaise à la Nouvelle-Guinée en 1912 et 1913 sous les auspices de A. Franssen Herderschee. Zoologie 8: 21–196 [plates IV–VIII].

[B51] Smith EA (1896) List of land-shells of the islands of Batchian, Ternate, and Gilolo. Proceedings of the Malacological Society 2: 120–122. 10.1093/oxfordjournals.mollus.a064658

[B52] Sowerby GB (1842) Thesaurus Conchyliorum, on Figures and descriptions of shells, Part 1. Containing monographs of the genera Helicina, Pupina, Rostellaria, Aporrhais, Struthiollaria, and Strombus. Sowerby, Great Russell Street, London, 1–39.

[B53] Sykes ER (1904) On some non-marine shells from the Austro- and Indo-Malayan regions. Journal of Malacology 11(1): 87–92.

[B54] Tapparone Canefri C (1883) Fauna malacologica della Nuova Guinea e delle isole adiacenti. Parte 1. Molluschi estramarini. Annali di Museo civico di storia naturale 19: 5–313.

[B55] Tapparone Canefri C (1886) Fauna malacologica della Nuova Guinea e delle isole adiacenti. Parte 1. Molluschi estramarini. Supplemento I. Annali di Museo civico di storia naturale 2(4): 113–200.

[B56] Tryon GW (1886) Manual of Conchology, structural and systematic with illustrations of the species, vol. II Zonitidae. Academy of Natural Sciences, Philadelphia, 256 pp.

[B57] Valdez BK Parcon JA de Chavez ERC (2021) Malacofaunal diversity and distribution in the Masungi Georeserve in Luzon Island, Philippines. Philippine Science Letters 14(1): 29–50.

[B58] van Benthem Jutting WSS (1959) Non- marine mollusca of the North Mollucan Island Halmahera, Ternate, Batjan, and Obi. Treubia 25(1): 25–87.

[B59] Vermeulen JJ Liew TS (2022) Land snails and slugs of Sabah and Labuan (Malaysia). Institute for Tropical Biology and Conservation, Universiti Malaysia Sabah, Kota Kinabalu, Malaysia, [iii–x] 1–432.

[B60] Vermeulen JJ Liew TS Schilthuizen M (2015) Additions to the knowledge of the land snails of Sabah (Malaysia, Borneo), including 48 new species. ZooKeys 531: 1–139. 10.3897/zookeys.531.6097 PMC4668911 26692803

[B61] von Martens E (1863) Cyclostomacea in insulis Moluccis proprie sit dictis nec non ins. Halmahera (Djilolo) lecta et breviter descripta. Malakozoologische Blätter 10: 83–87.

[B62] von Martens E (1864) Diagnosen neuer Arten von Heliceen aus dem indischen Archipel. Monatsberichte der Königlichen Preussischen Akademien der Wissenschaften zu Berlin 1864: 264–270.

[B63] von Martens E (1867) Die Landschnecken. Die Preussische expedition nach Ost-Asien: nach amtlichen quellen. Zoologischer Teil. Zweiter Band. Königliche Geheime Ober-Hofbuchdruckerei, Berlin, 447 pp.

[B64] von Martens E (1873) Ueber Landschnecken aus Celebes. Malakozoologische Blätter 20: 155–177.

[B65] Wallace AR (1869) The Malay Archipelago. Periplus Editions, Singapore, 512 pp.

[B66] Wallace AR Adams H (1865) List of the land shells collected by him in the Malay Archipelago: with Descriptions of the new species by Mr. Henry Adams. Proceedings of the scientific meetings of the Zoological Society of London 33(1): 405–416. 10.1111/j.1469-7998.1865.tb02358.x

